# The DCDC2/ENO1 axis promotes tumor progression and immune evasion in intrahepatic cholangiocarcinoma via activating FGL1-LAG3 checkpoint

**DOI:** 10.1186/s13046-025-03436-1

**Published:** 2025-06-18

**Authors:** Wenze Wan, Yuan Li, Wentao Sun, Zewei Cheng, Fen Ma, Sheng Shen, Houbao Liu, Jiwei Zhang

**Affiliations:** 1 Department of Biliary Surgery, Zhongshan Hospital, Fudan University, Shanghai, 200032 China; 2 Shanghai Key Laboratory of Compound Chinese Medicines, The MOE Key Laboratory for Standardization of Chinese Medicines, Institute of Chinese Materia Medica, Shanghai University of Traditional Chinese Medicine, Shanghai, 201203 China; 3 Department of Gastrointestinal Surgery, Zhongnan Hospital of Wuhan University, Wuhan, 430000 China; 4 Biliary Tract Disease Institute, Fudan University, Shanghai, 200032 China; 5 Shanghai Biliary Tract Minimal Invasive Surgery and Materials Engineering Research Center, Shanghai, 200032 China

**Keywords:** Intrahepatic Cholangiocarcinoma, Tumor-associated antigen, Progression, Immune evasion, Doublecortin domain containing 2, Enolase 1, Fibrinogen-like protein 1

## Abstract

**Background & Aims:**

ICC is a malignant tumor that originates from the intrahepatic bile ducts with insidious symptoms and a poor prognosis. Early diagnosis methods and therapeutic targets are urgently needed for ICC.

**Methods:**

We utilized a comprehensive set of analytical techniques to elucidate the role and mechanisms of DCDC2 in ICC. Our study included protein microarrays, transcriptome analysis, functional assays, immunofluorescence, dual-luciferase reporter assays, as well as xenograft models and humanized PBMC models.

**Results:**

Our study demonstrates that elevated levels of anti-DCDC2 autoantibodies in the serum of ICC patients indicate its potential utility as a diagnostic biomarker. Comprehensive in vitro and in vivo analyses reveal that DCDC2 promotes ICC proliferation, metastasis, and immune evasion. Mechanistically, DCDC2 stabilizes ENO1, resulting in enhanced AKT phosphorylation and increased expression of FGL1. Notably, elevated FGL1 levels significantly impair CD8^+^ T cell functionality via the FGL1-LAG3 axis.

**Conclusion:**

Our findings position anti-DCDC2 autoantibody as a promising diagnostic biomarker for ICC, associated with poor prognostic outcomes, and elucidate its critical role in tumor growth and immune evasion through its interaction with ENO1.

**Supplementary Information:**

The online version contains supplementary material available at 10.1186/s13046-025-03436-1.

## Introduction

Cholangiocarcinoma (CCA) is a malignant tumor that originates from the epithelium of the bile ducts. It can be further classified into intrahepatic cholangiocarcinoma (ICC), perihilar duct cholangiocarcinoma (PHCC), and distal duct cholangiocarcinoma (DCC). PHCC and DCC are also referred to as extrahepatic cholangiocarcinoma (ECC) [1]. ICC is the second most common cause of primary liver cancer [2, 3]. The symptom of ICC in the initial stage is not apparent, and 60–70% of patients do not have the opportunity for surgery [1, 4, 5]. On the other hand, ICC is not sensitive to chemoradiotherapy, and the median overall survival (OS) after first-line treatment is only about 1 year, and only 6 months after second-line treatment [6, 7]. Immunotherapy has improved the prognosis of a variety of tumors, but almost half of patients with ICC have low response rates to immune checkpoint inhibitors (ICIs) [8]. The immunosuppressive microenvironment of ICC promoting tumor immune evasion is one of the important causes for the poor therapeutic effect of immunotherapy in ICC [9]. Therefore, there is an urgent need to find more early diagnosis methods and therapeutic targets to improve the prognosis of patients with ICC.

In tumor patients, various factors, such as the loss of immune tolerance of tumor cells, inflammation, altered protein expression, and abnormal post-translational modification or localization of proteins, could lead to abnormal exposure or presentation of tumor-associated antigens (TAAs) [10–13]. The identification and study in TAAs will help us understand the physiological and pathological changes and mechanisms in the occurrence and development of tumors. It will also provide potential targets for targeted therapy. Tumor-associated autoantibodies (TAAbs) have been proposed as biomarkers in the diagnosis, monitoring of progression, and prediction of prognosis [14, 15].

DCDC2 (Doublecortin domain-containing 2) belongs to the Doublecortin (DCX) family, which is characterized by the presence of two DCX domains. DCX proteins are associated with neuronal migration during cortical development and are involved in nuclear translocation and the maintenance of bipolar morphology during cell migration [16]. DCX can also act as a platform for protein interactions, and interacts with a variety of proteins, including AP-1/2, JNK, and JIP [17–20]. DCDC2 is involved in tubulin and microtubule polymerization, thus affecting the development of primary cilia [21]. In the biliary system, a DCDC2 mutation is associated with abnormal development of primary cilia in the bile duct. This leads to dysregulation of bile duct homeostasis and the formation of"cytotoxic"bile, which could cause neonatal sclerosing cholangitis [22, 23]. However, the functional significance and mechanism of DCDC2 in ICC tumors are largely unknown.

In this study, we found that DCDC2 is an ICC-associated antigen. DCDC2 is abundantly expressed in ICC and is associated with a poor prognosis. Meanwhile, the anti-DCDC2 autoantibody may be used as a biomarker for early diagnosis of ICC. Moreover, DCDC2 promotes the proliferation, migration, and immune evasion of ICC cells by interacting with and stabilizing ENO1.

## Materials and methods

### Human samples

Serum from 3 healthy donors and 3 patients with CCA was used in the HuProt microarray detection. Twelve pairs of ICC and eleven pairs of ECC frozen tissues were collected for RNA-seq (Fudan-Cohort-I). Eight pairs of ICC and six pairs of ECC frozen tissues were collected for qPCR analysis of DCDC2 mRNA (Fudan-Cohort-II). Additionally, serum from 80 healthy donors, 14 cholelithiasis patients, 27 gallbladder cancer (GBC) patients, 38 ECC patients, and 92 ICC patients was collected from Zhongshan Hospital, Fudan University for ELISA (Fudan-Cohort-III). A total of 188 pairs of paraffin-embedded ICC samples were included in the tissue microarray (Fudan-Cohort-IV). All the tissues were obtained from Zhongshan Hospital, Fudan University. All sample donors signed informed consent for the use of biological samples and clinical information.

### Cell lines and cell culture

The human cholangiocarcinoma cell lines (HCCC-9810 and HuCC-T1) were cultured in RPMI-1640 medium supplemented with 10% FBS, 2 mM glutamine and 1% penicillin–streptomycin (P/S). HEK293T cells were cultured in DMEM High Glucose with 10% FBS and 2% P/S. HCCC-9810 and HuCC-T1 were purchased from COBIOER BIOSCIENCES Co., Ltd. (Nanjing, China). HEK293T cell line was obtained from the Cell Bank/Stem Cell Bank, Chinese Academy of Sciences. The sequences of shRNA, sgRNA and siRNA were as followed:

- shDCDC2#1: 5′ GTACTACAAATGGTCACAGAA 3′;
- shDCDC2#2: 5′ GCTGCAGCAGGTTAATAATGA 3′;
- shENO1#1: 5′ CGTGAACGAGAAGTCCTGCAA 3′;
- shENO1#2: 5′ GCATTGGAGCAGAGGTTTACC 3′;
- sgENO1: 5′ GGACTTCTCGTTCACGGCCTTGG 3′;
- siFGL1#1: 5′ CTGAACATATCCATGCGCAAT 3′;
- siFGL1#2: 5′ GACGATCTGATGGCAGTGAAA 3′;
- siFgl1#1: 5′ GTATGCAGATTGTTCAGAGAT 3′;
- siFgl1#2: 5′ CCATTGCTCTGATGATGGGAA 3′.

### Immunohistochemistry

IHC was conducted based on EnVision System. DCDC2 antibody (1:25 dilution; Santa Cruz, Cat No. sc-166051); ENO1 antibody (1:100 dilution; Proteintech, Cat No. 11204–1-ap); AKT1 antibody (1:100 dilution; Abclonal, Cat No. A17909); Phospho-AKT1 (S473) antibody (1:100 dilution; Abclonal, Cat No. AP0637); Phospho-AKT1 (T308) antibody (1:100 dilution; Abclonal, Cat No. AP1214); Ki67 antibody (1:500 dilution; Abcam, Cat No. ab15580) were used in IHC following the manufacturer’s protocols. The staining intensity was independently scored by two pathologists.

### Immunofluorescence

Immunofluorescence was performed based on the Tyramide signal amplification method. The following primary antibodies were used: DCDC2 antibody (1:25 dilution; Santa Cruz, Cat No. sc-166051); ENO1 antibody (1:100 dilution; Proteintech, Cat No. 11204-1 -ap).

### Western blot analysis

Cells were lysed using RIPA buffer supplemented with a protease or phosphatase inhibitor cocktail and denatured at 100 °C for 10 min in the loading buffer. Proteins were subjected to SDS-PAGE gel and transferred onto nitrocellulose or PVDF membrane. After blocking, the membranes were incubated with primary antibodies: GAPDH antibody (1:1000 dilution; Proteintech, Cat No. 60004-1 -Ig); DCDC2 antibody (1:1000 dilution; Abclonal, Cat No. A17704); ENO1 antibody (1:2000 dilution; Proteintech, Cat No. 11204-1 -ap); FGL1 antibody (1:1000 dilution; Abclonal, Cat No. A20335); AKT1 antibody (1:1000 dilution; Abclonal, Cat No. A17909); Phospho-AKT1 (S473) antibody (1:1000 dilution; Abclonal, Cat No. AP0637); Phospho-AKT1 (T308) antibody (1:1000 dilution; Abclonal, Cat No. AP1214); NEDD4L antibody (1:1000 dilution; Abclonal, Cat No. A9078); FBXW7 antibody (1:1000 dilution; Abclonal, Cat No. A5872); Ubiquitin antibody (1:1000 dilution; Abclonal, Cat No. A2129); Myc-Tag antibody (1:1000 dilution; Abclonal, Cat No. AE010); DDDDK-Tag antibody (1:1000 dilution; Abclonal, Cat No. AE005).

### Enzyme linked immunosorbent assay (ELISA)

The ELISA protocol for detecting anti-DCDC2 antibodies in plasma samples involves coating a 96-well plate with human DCDC2 recombinant protein diluted to 1 µg/ml in carbonate buffer, followed by overnight incubation at 4 °C. After washing, the plate is blocked with 5% non-fat dry milk in PBST for 1 h, then washed again. Plasma samples are added and incubated for 1.5 h, followed by washing and incubation with HRP-labeled goat anti-human IgG (diluted 1:3000). After washing, TMB substrate solution is added for 10 min, and the reaction is stopped with TMB stop solution. Absorbance is measured at 450 nm. Results show that anti-DCDC2 antibody levels are higher in cholangiocarcinoma patients compared to those with cholelithiasis, gallbladder cancer, and healthy controls. The recombinant protein used was from Proteintech (Catalog Number: Ag25577).

For detecting FGL1 concentration in supernatant of ICC cells, 5 × 10^5^ HCCC-9810 or HuCC-T1 cells were seeded into a six-well plate and cultured for 24 h. The cell supernatant is collected by centrifugation at 13,000 rpm for 10 min at 4 °C. The concentration of FGL1 was determined using Human Fibrinogen-like protein 1(FGL1) ELISA kit (CUSABIO, Catalog Number: CSB-EL008653HU).

### Quantitative real-time reverse transcription PCR (qPCR)

Total RNA was prepared using TRIzol reagent. RNA was reverse transcribed into cDNA. qPCR was conducted with SYBR Green Master. The mRNA expression was normalized using the 2-ΔΔCt method. Primer sequence for PCR amplification are as follows:

- DCDC2-F: 5′ CCAGCTTCTCGCCTCCTTATC 3′;
- DCDC2-R: 5′ GGCCTTCTCATCGTTGACTTG 3′;
- FGL1-F: 5′ GGGTCAAACAGCAACAGGTC 3′;
- FGL1-R: 5′ CTCCTCCATCGGACATGTCA 3′;
- GAPDH-F: 5′ GGAGCGAGATCCCTCCAAAAT 3′;
- GAPDH-R: 5′ GGCTGTTGTCATACTTCTCATGG 3′.

### Migration and invasion assay

A total of 10 × 10^4^ HCCC-9810 or 5 × 10^4^ HuCC-T1 cells was plated in the top chambers (24-well, 8 μm pore size, Corning) with serum-free medium for the migration assay. The bottom chambers were filled with 500 μL of complete medium. After incubated for 24 h, the chambers were fixed with 4% paraformaldehyde and then stained with 0.025% crystal violet. The cells in the upper chamber were gently wiped with a cotton swab. The membranes were photographed with an inverted microscope after being washed and dried.

### Cell proliferation assay

Cell proliferation was assessed using the Counting Kit-8 according to the manufacturer’s protocol. A total of 3000 HCCC-9810 or 1500 HuCC-T1 cells were seeded in 96-well plates and incubated with CCK8 for 2 h at 37 °C and 5% CO_2_. The absorbance was measured at 450 nm. For the colony formation assay, 800 cells were seeded into a six-well plate and cultured for 10 days. Then, the colonies were fixed with 4% paraformaldehyde for 15 min and stained with 0.025% crystal violet for 20 min.

### Coimmunoprecipitation assay

Cells were lysed using passive Cell Lysis Buffer with protease inhibitors on ice for 15 min. The proteins were incubated with 2 μg DCDC2 antibody (Abclonal, Cat No. A17704); ENO1 antibody (Proteintech, Cat No. 11204-1 -ap); Myc-Tag antibody (Abclonal, Cat No. AE010); DDDDK-Tag antibody (Abclonal, Cat No. AE005) and incubated at 4 °C overnight on a rotating shaker. Protein A/G agarose (30 μL) was added and incubated for 1 h at room temperature. The bound proteins were eluted by boiling in 2 × SDS sample buffer for 5 min at 100 °C.

### Dual-luciferase reporter assay

The sequence of FGL1 promoter and the corresponding mutants were cloned and inserted into pGL3.basic plasmids. 293-FT cells were cultured to approximately 70% confluence and co-transfected with luciferase reporter plasmids, internal control plasmids and vehicle or ENO1 overexpression vectors. After 48 h, the activity of luciferase was measured according to the manufacturer’s instructions. The ratio of firefly luciferase to renilla luciferase was calculated.

### Chromatin Immunoprecipitation (ChIP) and qPCR Analysis

Cells were fixed with 1% formaldehyde (1 × 10⁶ cells/mL, 5 min, 25 °C), quenched with 0.125 M glycine, and lysed in ice-cold buffer. Nuclei were digested with MNase (37 °C, 20 min), followed by sonication (3 cycles, 30 s ON/45 s OFF) to shear chromatin. Cleared lysates were incubated overnight at 4 °C with target-specific antibodies and Protein G beads, then washed with gradient buffers. DNA–protein complexes were eluted (1% SDS, 0.1 M NaHCO₃) and purified using silica columns. For qPCR, ChIP DNA was amplified with SYBR Green Master Mix (95 °C/5 min; 40 cycles: 95 °C/15 s, 60 °C/30 s, 72 °C/30 s) using primers flanking binding sites. Fold-enrichment was calculated via ΔΔCt method normalized to input DNA, with IgG and no-template controls.

- FGL1-F: 5′ AAGTTACAGTACAAAAATTCTCT 3′;
- FGL1-R: 5′ CCAATACTCTGATATTTTTCTTT 3′;
- MYC-F: 5′ TCCTGCCTCGAGAAGG 3′;
- MYC-R: 5′ GGATCAAGAAAAAGACATTAAGG 3′.

### Flow cytometry

Freshly excised tumor tissues were collected to isolate tumor-infiltrating lymphocytes (TILs). Briefly, the tumor tissues were dissected and transferred into RPMI medium. Mechanical disruption of the tissues was performed, followed by digestion in a mixture of 0.3 mg/ml DNase I (Sigma-Aldrich) and 0.25 mg/ml Liberase TL (Roche) in serum-free RPMI medium at 37 °C for 30 min in a CO_2_ incubator. The digested tissues were then dispersed through a 40 μm cell strainer (BD Biosciences) to remove tissue clumps. The single cells were washed and suspended in Hank's Balanced Salt Solution (HBSS) containing 1% FBS for staining and flow cytometric analysis. For the splenic tissue, it was homogenized directly followed by treatment with red blood cell lysis buffer. For cytokine detection, treat the cells with Cell Activation Cocktail (Biolegend, 423,303) at a temperature of 37 °C for a duration of 6 h in a CO_2_ incubator.

The dissociated tumor and splenic tissue, as well as the digested cells, were subjected to staining in the dark. The diluted cell surface antibody mixture (1:100) was incubated with the samples at a temperature of 4 °C for 30 min. The samples then were washed and fixed with the fixation solution at 4 °C for 30 min. The samples and the intracellular antibody mixture (1:100) were incubated on ice for 45 min for intracellular staining. Following antibody staining, wash the cells and resuspend them in suspension for flow cytometry analysis. Flowjo software was used for data analysis.

### Xenograft model

For the subcutaneous xenograft model, 5 × 10^6^ HuCC-T1 cells were injected into the flanks of female mice (BALB/c nude, 5 weeks old, *n* = 5 per group). Tumor volumes were measured with a caliper every 4 days and calculated using the formula V = (length × width^2^)/2. The mice were sacrificed after four weeks. For the liver metastasis model, 2 × 10^6^ HuCC-T1 cells expressing luciferase were injected into the spleen of 4-week-old female BALB/c nude mice (*n* = 5 per group). D-Luciferin sodium salt was used for in vivo imaging at 1, 3, and 5 weeks after injection, following the manufacturer’s protocols.

For the subcutaneous syngeneic xenograft model, 1 × 10^6^ SB-1 cells were injected into the flank of 6-week-old female C57 mice (*n* = 5 per group). Once the tumors reached approximately 100 mm^3^, LAG-3 antibody (Selleck, clone: C9B7W, 200 μg/100 μL per mouse) was administered via intraperitoneal injection, with treatments occurring every three days for a total of three cycles. Tumor volume was measured using calipers every three days, and calculated using the formula V = (length × width^2^)/2. After 20 days, the mice were euthanized, and tissues were collected for subsequent experiments.

### Co-culture model of PBMCs and SB-1 cells

One day before the experiment, PBMCs were incubated overnight in the presence of CD3/CD28 (Stermcell, 100–1572) in the culture medium. SB-1 cells were collected and adjusted to 5.0 × 10^5^ cells/mL. In a 24-well plate, 0.2 mL of SB-1 cell suspension was mixed with 0.2 mL of PBMCs (5.0 × 10^6^ cells/mL). For the treatment group, LAG-3 antibody was added to a final concentration of 2 µg/mL (Selleck, clone: C9B7W). The cells were incubated for 48 h, followed by collection for flow cytometry analysis.

### Humanized mouse xenograft model

Humanized mouse xenograft model was constructed according to protocols approved by the IMMUCAN Biote. Co., LTD (IMMUCAN) Institutional Animal Care and User Committee (IACUC). Briefly, female NOG mice (5–6 weeks old, Beijing Vital River Laboratory Animal Technology Co., Ltd.) underwent myeloablation with busulfan (25 mg/kg, i.p.; B2635, Sigma-Aldrich, St. Louis, MO, USA) one week prior to tumor cell transplantation. HuCC-T1 cells in logarithmic growth phase were harvested and 1 × 10^6^ cells in 100 μL mixed in a 1:1 high-concentration matrix were subcutaneously implanted in the dorsal region of NOG mice. The infusion of PBMCs (5 × 10^6^ cells/mouse, i.v. Schbio, PBMNC050C) was performed on day 7. For the α-LAG3 (MCE, Relatlimab, 1,673,516– 98-7) treatment model, when the tumor volume reached approximately 100 mm^3^, mice were randomized into groups for α-LAG3 treatment every three days, totaling four treatments. The engraftment levels of hCD45^+^ cells were determined 2 weeks post-PBMCs transplantation by flow cytometric quantification of peripheral blood CD45^+^ cells. Tumors were measured with calipers every 3 days, and volumes (in mm^3^) were calculated using the formula (length × width^2^)/2.

### Statistical analysis

The experiment in vitro was run in triplicate. The experiment in vivo was run in triplicate. Data were analyzed using GraphPad Prism 9 software. The student’s t-test was applied to determine the significance of differences between normally distributed data. The Mann–Whitney test was used for non-normally distributed data. Overall survival was assessed using the Kaplan–Meier method and the log-rank test. All the tests are two-tailed.

## Results

### DCDC2 is an ICC-associated antigen and correlates with a poor prognosis

**Fig. 1:**
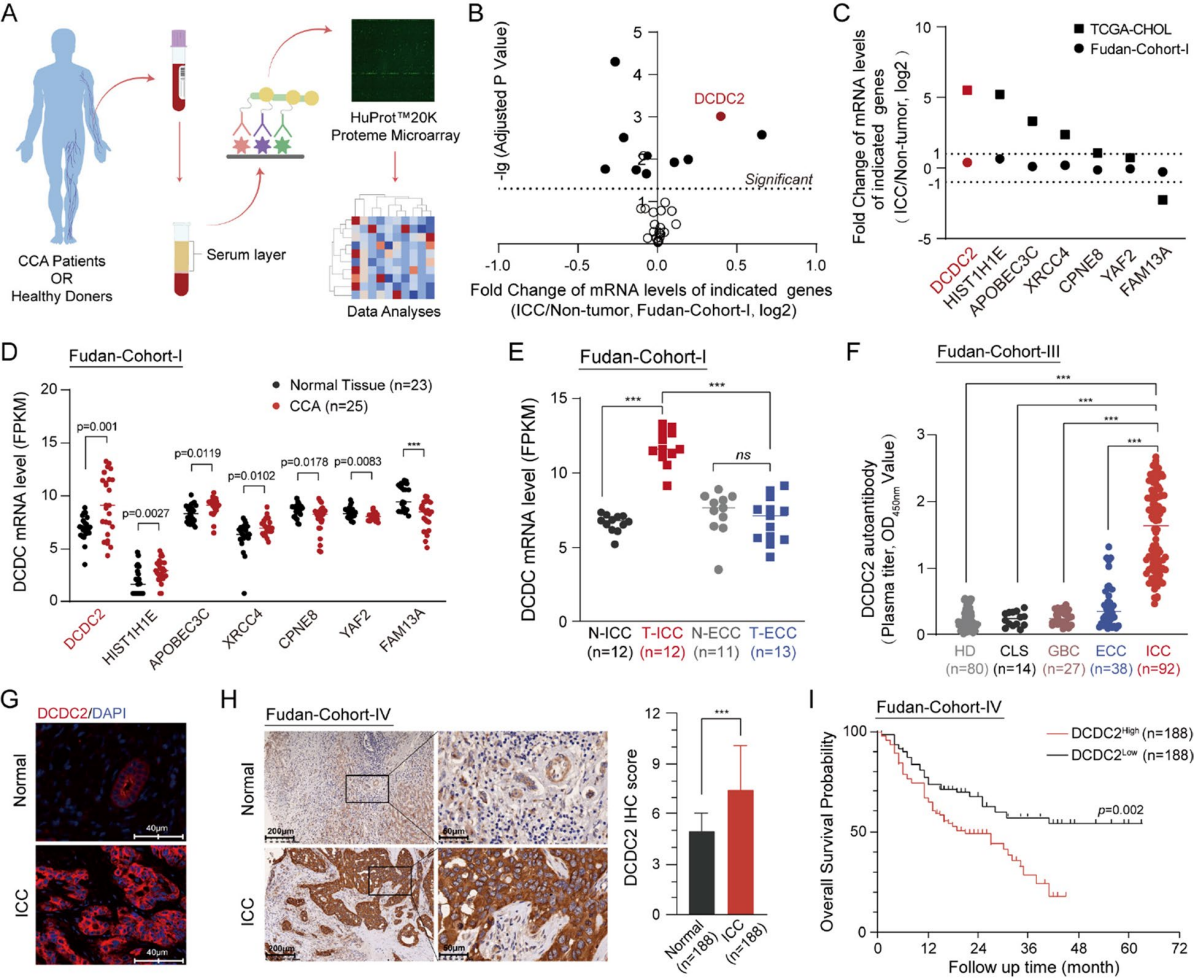
DCDC2 is an ICC-associated antigen and is correlated with a poor prognosis. **A** Flow chart of the HuProt microarray analysis. **B** Volcano plot of TAAbs corresponding genes in the FUDAN cohort. **C** Fold change of mRNA level of the 7 differentially expressed genes in the TCGA-CHOL and Fudan-Cohort-I databases. **D** Expressions of the 7 genes in the FUDAN cohort. **E** The Expressions of DCDC2 in ICC and ECC in the Fudan-Cohort-I. **F** Plasma titer of DCDC2 autoantibody detected by ELISA in Fudan-Cohort-III. **G** The Representative IF image of DCDC2 in Fudan-Cohort-IV. **H** The representative IHC image of DCDC2 Fudan-Cohort-IV. Quantitative analysis was shown in the graphs. **I** The overall survival of Fudan-Cohort-IV with low or high DCDC2 expression is shown based on staining intensities in (I). ***p < 0.001

To investigate CCA-associated antigens and autoantibodies, we utilized the HuProt microarray to detect autoantibodies in samples obtained from three patients (one case of ICC and two ECC patients) as well as three healthy donors (Fig. 1A). Data analysis revealed that there were 31 TAAbs in the serum from CCA patients (Supplementary Fig. 1 A). The expression levels of the 28 corresponding genes which encoded these 31 TAAbs were analyzed in the FUDAN cohort-I RNA-seq data and the TCGA-CHOL cohort (Supplementary Table 1 and Supplementary Table 2). Among these 28 genes, 10 genes were significantly deregulated in CCA tissues compared with the corresponding non-tumor tissues, both in the FUDAN cohort-I and TCGA-CHOL cohort (Fig. 1B and Supplementary Fig. 1B). Besides the top-ranked fold change (CCA/Non-tumor) of DCDC2 expression levels in these two cohorts (Fig. 1C), the mRNA levels of DCDC2 of CCA tissues were also in remarkably high levels (Fig. 1D and Supplementary Fig. 1 C). Moreover, DCDC2 mRNA levels of the ICC and ECC tissues from Fudan cohort-I and TCGA-CHOL cohort were analyzed in detail, and it was in ICC tissues that DCDC2 mRNA levels were specifically upregulated (Fig. 1E and Supplementary Fig. 1D). Similar results were found in another independent FUDAN cohort-II (Supplementary Fig. 1E). Furthermore, DCDC2 showed tissue-specific high expression in ICC tissues, among all the normal tissue types (GTEx database) and pan-cancer types (TCGA database, Supplementary Fig. 1 F). Moreover, the existence of DCDC2 autoantibody was examined in Fudan cohort-III, consisted of the serum from healthy donors (HD), patients with cholelithiasis (CLS), with gallbladder cancer (GBC), with ECC, or with ICC. Consistent with the results that were found in HuProt microarray, the levels of DCDC2 autoantibody were significantly higher in ICC samples (Fig. 1F).

Next, immunofluorescence (IF) and immunohistochemical staining (IHC) assays were performed to examine the expression and distribution of the DCDC2 protein in ICC and Non-tumor tissues. The IF results showed that the low-leveled DCDC2 protein was primarily concentrated in the cell membrane within normal bile duct. However, in ICC tissues, the expression of the DCDC2 protein was much higher and its distribution was more irregular (Fig. 1G). Similar results were found in IHC assay in the samples from Fudan-cohort IV (Fig. 1H). Moreover, high expression levels of DCDC2 were correlated with poorer prognosis in the ICC patients of this cohort (Fig. 1I and Supplementary Fig. 1G). Collectively, DCDC2 is frequently over-expressed in ICC and serves as an ICC-distinctive antigen, which may both be used as biomarkers for the diagnosis of ICC, acompanying with its priority in prognosis prediction for ICC.

### DCDC2 promotes ICC progression by activating AKT pathway

**Fig. 2:**
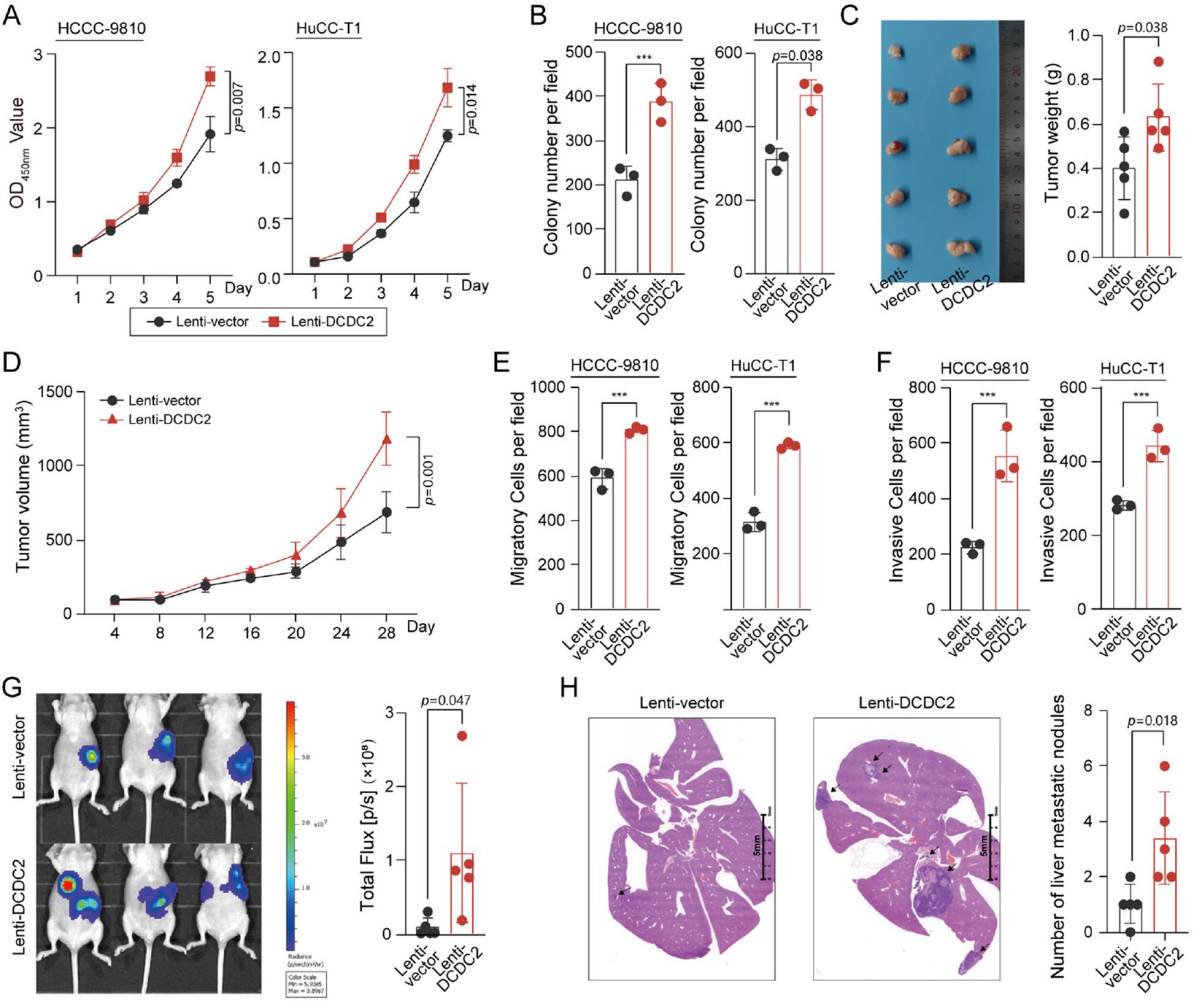
DCDC2 promotes ICC proliferation and metastasis. **A** CCK-8 assays showed that ectopic expression of DCDC2 promoted the proliferation ability of the ICC. **B** Colony formation assays showed that ectopic expression of DCDC2 promoted the colony formation of the ICC. **C** Tumor growth curves of nude mice inoculated with the vehicle, and DCDC2 overexpressed HuCC-T1 cells. **D** Tumor weight of nude mice inoculated with the vehicle, and DCDC2 overexpressed HuCC-T1 cells. **E** Transwell migration assays showed that overexpression of DCDC2 promoted the migration abilities of ICC cells. **F** Transwell invasion assays showed that overexpression of DCDC2 promoted the invasion abilities of ICC cells.** G** In vivo imaging (left) and quantitative analyses (right) of liver metastasis 5 weeks after injection. **H** Representative H&E staining image (left) and quantitative analyses (right) of liver metastasis node. ***p < 0.001

Then we tried to further investigate the biological roles of the upregulated DCDC2 in ICC. We tested the expression of DCDC2 in CCA cell lines (Supplementary Fig. 2 A). DCDC2 was over-expressed in HCCC-9810 and HuCC-T1 cell lines via Lenti-virus system to investigate its function and mechanisms (Supplementary Fig. 2B), nominated as Lenti-DCDC2 for the stable cell lines. DCDC2 promoted the proliferation rate of ICC cells in vitro (Fig. 2A), and also enhanced the colony formation ability of these cells (Fig. 2B and Supplementary Fig. 2 C). In vivo assays confirmed the promoting effects of DCDC2 on the tumorigenicity of ICC cells (Fig. 2C and D), with both increased tumor volumes and tumor weight. On the other hand, DCDC2 facilitated the metastasis of ICC cells in vitro and in vivo. Transwell assays showed that the migratory and invasive cells were increased in the Lenti-DCDC2 group (Fig. 2E, F, Supplementary Fig. 2D and E). To further evaluate the promoting effects of DCDC2 on ICC metastasis, a liver metastasis mouse model was established by injecting indicated HCCC-9810 cells into the spleen of nude mice. Bioluminescence imaging showed that DCDC2 promoted the metastasis of ICC cells in vivo (Fig. 2G). H&E staining of the mouse liver confirmed the increased metastatic loci of ICC cells (Fig. 2H).

**Fig. 3:**
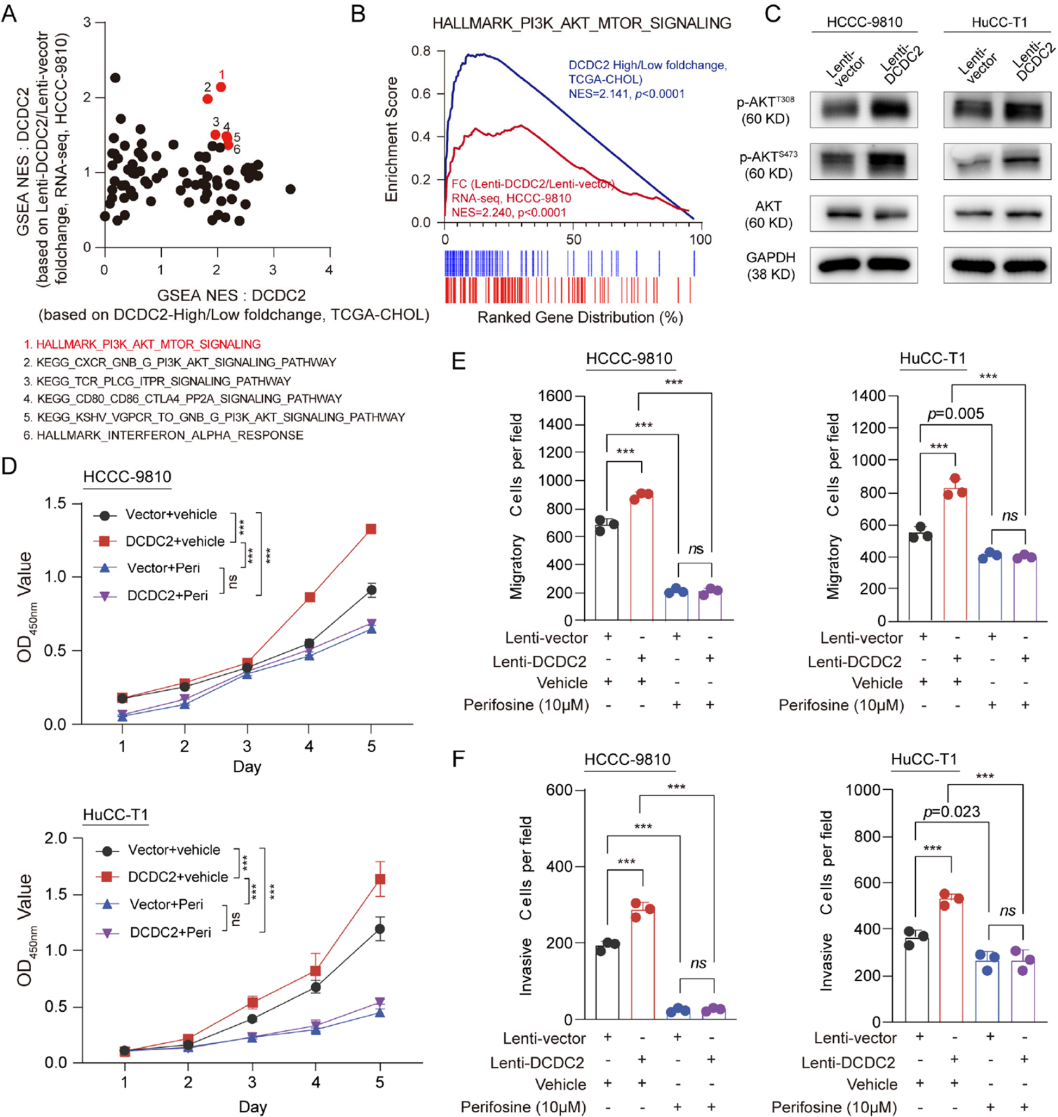
DCDC2 executed its oncogenic functions by AKT activation. **A** GSEA enrichment analysis was conducted on the differential genes in the group with high DCDC2 expression compared to the group with low DCDC2 expression. Simultaneously, GSEA enrichment analysis was performed on the differential genes in the Lenti-DCDC2 group compared to the Lenti-vector group in the HCCC-9810 RNA-seq database.** B** GSEA was conducted on the differential genes between the high and low DCDC2 expression groups in the TCGA-CHOL dataset and HCCC-9810 RNA-seq database, focusing on the enrichment scores for the PI3K-AKT signaling pathway. **C** The protein levels of p-AKT^T308^, p-AKT^S473^, and AKT in ICC cells overexpressing DCDC2 were assessed by Western blotting.** D** The CCK-8 assay was used to evaluate the proliferation of ICC cells treated with Perifosine (Peri, 10 μM). **E** Quantitative analyses of transwell migration assay in ICC cells treated with Peri (10 μM). **F** Quantitative analyses of transwell invasion assay in ICC cells treated with Perifosine (10 μM). ***p < 0.001

To investigate the molecular mechanism by which DCDC2 exhibits its promoting effects on ICC progression, the RNA-seq dataset of Xenograft derived from Lenti-vector/Lenti-DCDC2 stable cells was analyzed by Gene Set Enrichment Analysis (GSEA), as well as the dataset of TCGA-CHOL classified by DCDC2-High or DCDC2-Low expression was also analyzed. The results showed that high-leveled DCDC2 significantly activated the PI3K-AKT signaling in ICC cells (Fig. 3A), and the top-scored biological process included Hallmark PI3K-Akt-MTOR signaling (Fig. 3B), KEGG CXCR-GNB/PI3K-Akt signaling pathway and KEGG KSHV vGPCR to GNB/G-PI3K-Akt signaling pathway. The activation of PI3K-AKT signaling pathway was confirmed by western blot, which showed that overexpression of DCDC2 promoted AKT phosphorylation in HCCC-9810 and HuCC-T1 cells (Fig. 3C). To investigate the contribution of PI3K-AKT signaling on the DCDC2-promoted ICC proliferation and metastasis, perifosine [24], an AKT phosphorylation inhibitor, was employed. CCK8 assays showed that when Akt was inhibited by perifosine, DCDC2 overexpression is unable to promote proliferation or migration (Fig. 3D). Consistently, transwell assays showed that perifosine also eliminated the promoted migration and invasion of DCDC2-overexpressed ICC cells (Fig. 3E, F, Supplementary Fig. 3 A and 3B).

### DCDC2 upregulates FGL1 to promote ICC immune evasion

**Fig. 4:**
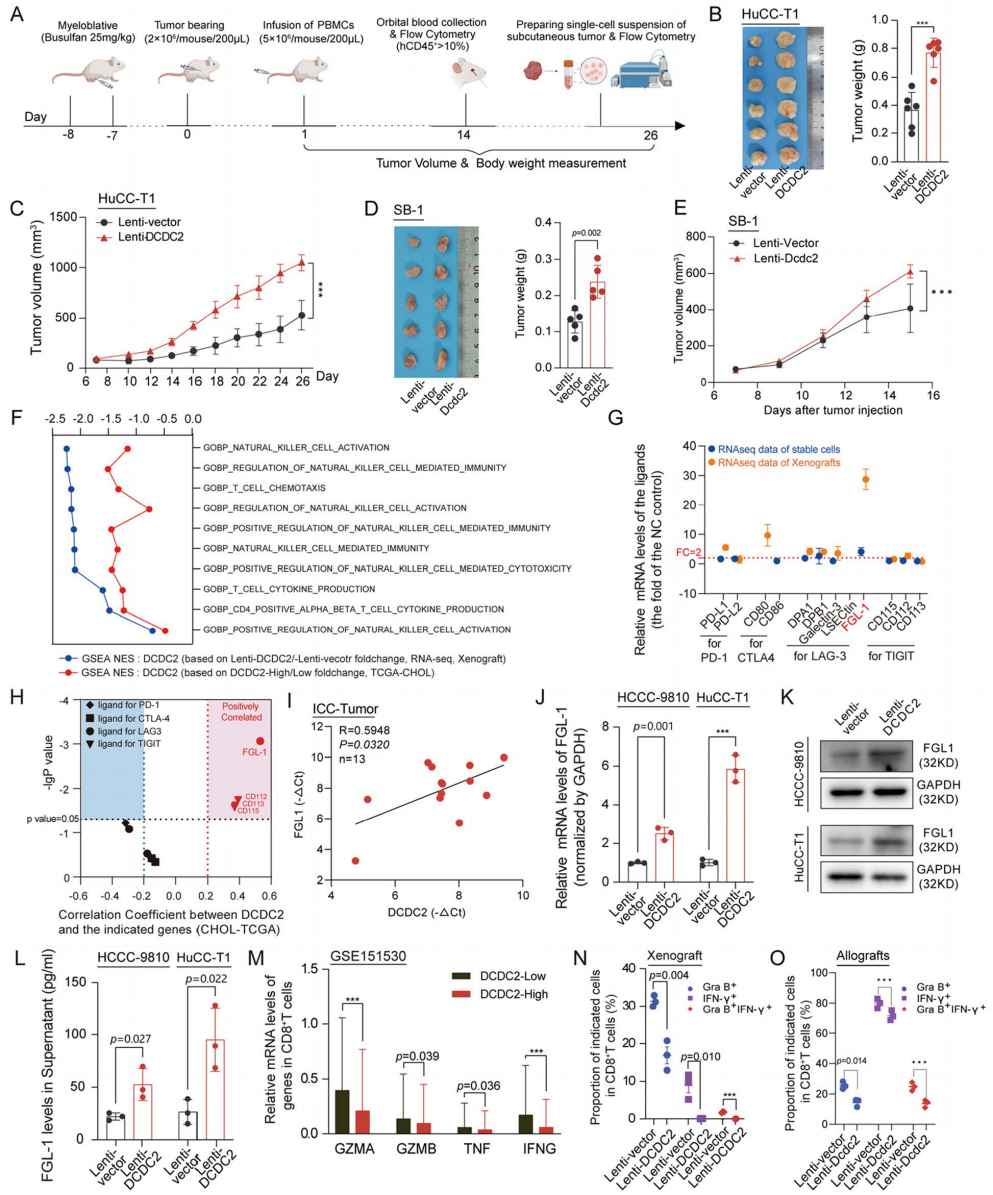
DCDC2 promotes ICC immune evasion and upregulates FGL1 expression. **A** Schematic diagram of humanized mouse xenograft model. **B** Overexpression of DCDC2 significantly promoted tumor weight of humanized mice. **C** Tumor growth curves of humanized mice showed overexpression of DCDC2 significantly promoted tumor growth. **D** Overexpression of Dcdc2 significantly promoted tumor weight of SB-1 derived syngeneic tumor. **E** Tumor growth curves of syngeneic model showed overexpression of Dcdc2 significantly promoted tumor growth. **F** Analysis of GSEA scores for immune-related pathways in the high DCDC2 expression group versus the low DCDC2 expression group using TCGA-CHOL and xenograft RNA-seq data. **G** Expression analysis of immune checkpoint ligands in tumor cells with high DCDC2 expression in the HCCC-9810 RNA-seq and xenograft RNA-seq databases. **H** Correlation analysis between DCDC2 and immune checkpoint ligands in the TCGA-CHOL database. **I** The expression of DCDC2 and FGL1 was positively correlated in human ICC tumors. **J** The relative expressions of FGL1 mRNA in ICC cells were assessed by qPCR after DCDC2 overexpression. **K** The protein levels of FGL1 in ICC cells were assessed by western blot after DCDC2 overexpression. **L** The protein levels of FGL1 in supernatant of ICC were assessed by ELISA after DCDC2 overexpression. **M** The relative expressions of GZMA, GZMB, TNF, IFNG of CD8^+^ T cells in scRNA-seq data. **N** The expressions of granzyme B and IFN-γ in CD8^+^ T cells in xenograft tumors of humanized mice were assessed by flow cytometry. **O** The expressions of granzyme B and IFN-γ in CD8^+^ T cells in allograft tumor of syngeneic model were assessed by flow cytometry. ***p < 0.001

AKT is a well-established classical pathway known to be involved in immune regulation. In the GSEA of the RNA-seq dataset of Xenograft and dataset of TCGA-CHOL, several classical immune related pathways were also enriched, such as KEGG TCR-PLCG-ITPR signaling pathway, KEGG CD80/CD86-CTLA4-PP2A signaling pathway, HALLMARK INTERFERON ALPHA RESPONSE, suggesting that the anti-tumor immunity function might be involved in DCDC2-mediated ICC progression (Fig. 3A). Thus, we also explored whether DCDC2 could influence tumor immunology. In vivo assays were designed in a humanized mouse xenograft model (Fig. 4A), with an effective engraftment level of hCD45^+^ cells at 2 weeks post-PBMCs transplantation (Supplementary Fig. 4 A). Consistently, DCDC2 significantly promoted both the volume and the weight of ICC tumor that generated in the humanized mice (Fig. 4B and C). We further utilized a mouse ICC cell line (SB-1) to establish a syngeneic model to verify the function of DCDC2. The results showed that DCDC2 promoted the volume and the weight of ICC in the syngeneic model. (Fig. 4D and E).

To explore the mechanism of DCDC2 promoting ICC immune evasion, we performed single-cell RNA sequencing (scRNA-seq) analysis using data from 3 cases of DCDC2 high expression and 3 of low expression in GSE151530. There were no significant differences in the proportions of T cells, B cells, TAMs, CAFs, and TECs between DCDC2 high and low cases (Supplementary Fig. 4B-4D). Further, we found that the proportions of T cell subsets in DCDC2 high and low cases were also comparable (Supplementary Fig. 4E-4G). Similar results were observed in TAMs and CAFs (Supplementary Fig. 4E-4G). We also analyzed the proportion of immune cells in tumors of the syngeneic model. The results showed that the overexpression of DCDC2 did not affect the proportion of immune cells (Supplementary Fig. 4H). Further GO enrichment analysis on the bulk RNA-sequencing data of the xenografts from the humanized mouse model showed that several immuno-potentiating pathways were negatively regulated, which were involved in natural killer cell activation, T cell activation and the related cytokine production (Fig. 4F). Correspondingly, the mRNA levels of the classic ligands in immune checkpoint were analyzed in the RNA-sequencing data of DCDC2-overexpressed HCCC-9810 cell line and the RNA-sequencing data of the DCDC2-overexpressed xenografts from the humanized mouse model, and it was FGL1 that was significantly upregulated in both these datasheets (Fig. 4G). Furthermore, the strong positive correlation between the mRNA levels of DCDC2 and FGL1 were observed in the TCGA-CHOL cohort (Fig. 4H). We collected 13 ICC tumor samples and performed qPCR to evaluate the expression of DCDC2 and FGL1, alongside an analysis of their correlation. The results showed that the expression of DCDC2 and FGL1 was positively correlated in ICC tumors (Fig. 4I). These results suggested that DCDC2 might influence tumor immunity through regulating a downstream effector FGL1.

FGL1 was previously identified as a major immune inhibitory ligand of LAG-3 [25]. The interaction of FGL1 and LAG-3 could suppress the function of CD8^+^ T cells and promote immune evasion independent of PD-1/PD-L1. The levels of FGL1 in DCDC2-overexpressed ICC cells were remarkably enhanced in mRNA levels (Fig. 4J), in intracellular protein levels (Fig. 4K), and in supernatant (Fig. 4L), drawing forth the hypothesis that FGL1-regulating CD8^+^ T cells might be deeply involved in the DCDC2-mediated anticancer immune response in ICC. The levels of several cytotoxic cytokines of CD8^+^ T cells were analyzed in GSE151530 datasheet, such as Granzyme A, Granzyme B, TNFα and IFN-γ. And as expected, the levels of these cytotoxic cytokines were much lower in the DCDC2-high group than those in the DCDC2-low group (Fig. 4M). In vivo assays further confirmed that CD8^+^ T cells within both xenografts and allografts from the Lenti-DCDC2 group exhibited reduced levels of Granzyme B and IFN-γ (Fig. 4N, O, supplementary Fig. 4I and 4 J), suggesting the suppressed function of CD8^+^ T cells in these tissues. Collectively, these results demonstrated that DCDC2 promotes ICC immune evasion through upregulating FGL1 and the subsequent inhibition of CD8^+^ T cells.

### ENO1 interacts with DCDC2 and contributes to the oncogenic function of DCDC2

**Fig. 5:**
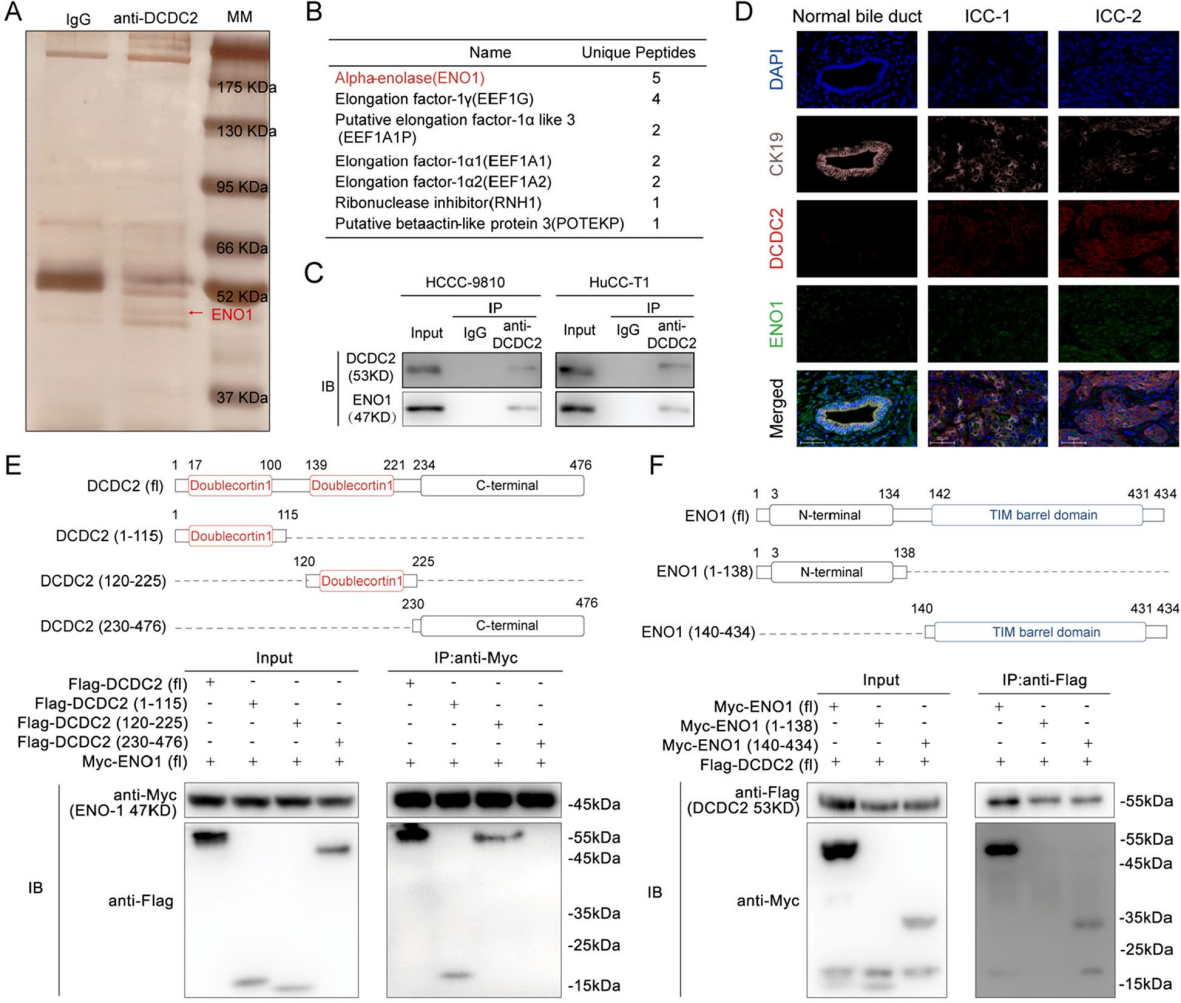
DCDC2 interacts with ENO1. **A** Molecules (mostly proteins) after Co-IP were visualized by silver staining following electrophoresis. The white arrow demonstrated the specific band for MS. **B** The list of proteins detected after MS. **C** Co-IP and Western blot verified the interaction of DCDC2 and ENO1. **D** Representative Immunofluorescence image of DCDC2 and ENO1 in human ICC tissue. **E** HEK-293 T cells were co-transfected with the plasmids encoding Flag-DCDC2, Flag-DCDC2 1–115, Flag-DCDC2 120–225, Flag-DCDC2 230–476, and Myc-ENO1. Protein was immunoprecipitated using an Anti-Myc antibody. **F** HEK-293 T cells were co-transfected with the plasmids encoding Myc-ENO1, Myc-ENO1 1–138, Myc-ENO1 140–434 and Flag-DCDC2. Protein was immunoprecipitated using an Anti-Flag antibody

Based on the results above, we demonstrated that DCDC2 promotes ICC progression by activating AKT pathway. Meanwhile, DCDC2 can promote ICC immune evasion through upregulating FGL1 and the subsequent inhibition of CD8^+^ T cells. Next, we attempt to determine the molecular mechanisms underlying the oncogenic function of DCDC2. It has been reported that the DCX domain, which exists two conservative sequence copies in DCDC2 protein, can act as a platform for protein interaction [19, 20]. Thus, we hypothesize that DCDC2 may function through interacting with other proteins. A Co-IP/Mass Spectrometry (MS) analysis was conducted using anti-DCDC2 antibody in ICC tissues (Fig. 5A). Among the identified proteins that interacted with DCDC2, ENO1 was high-ranked according to the number of scoring unique peptides (Fig. 5B). The interaction between ENO1 and DCDC2 was further confirmed in HCCC-9810 and HuCC-T1 cells (Fig. 5C and supplementary Fig. 4 K). Furthermore, these two proteins were co-localized in ICC tissues (Fig. 5D). Next, truncated DCDC2 and ENO1 constructs were used to map the domains necessary for the interaction. Co-IP assays showed that the first DCX domain of DCDC2 and the TIM barrel domain of ENO1 were crucial for binding with each other (Fig. 5E and F).

**Fig. 6:**
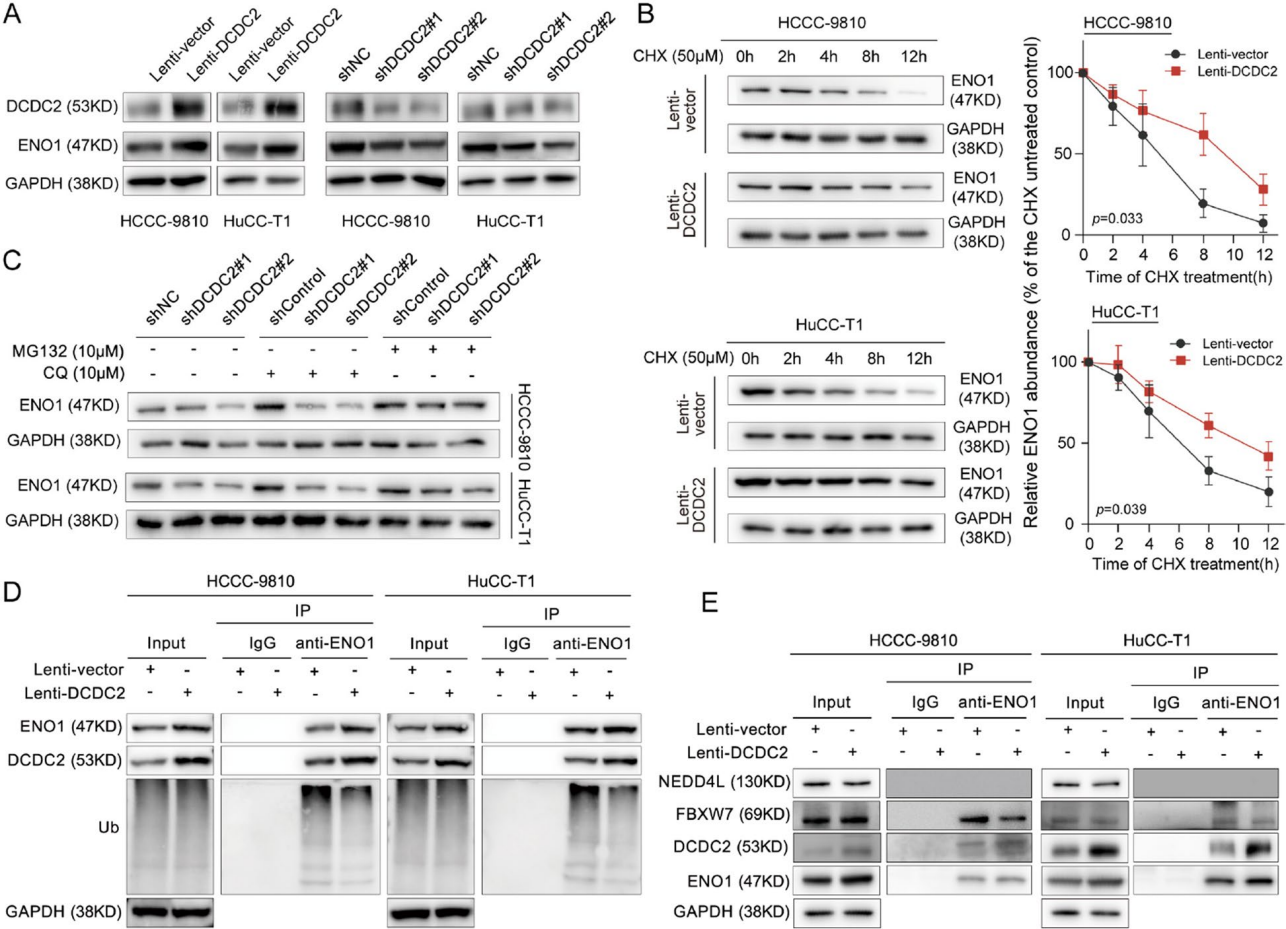
DCDC2 stabilized ENO1 by suppressing its ubiquitination. **A** The protein levels of DCDC2 and ENO1 were assessed by western blot after DCDC2 overexpression (left) or knockdown (right). **B** Overexpression of DCDC2 stabilizes ENO1. Cells were treated with 50 μM cycloheximide (CHX) for 0, 2, 4, 8, and 12 h, and the protein levels of ENO1 were assessed by western blot. **C** MG-132, but not CQ, partially rescued the decrease of ENO1 in DCDC2 knockdown cells. Cells were treated with 10 μM CQ or MG-132 for 8 h, and the protein levels of ENO1 were assessed by western blot. **D** The ubiquitination of ENO1 in cells overexpressing DCDC2 was detected by Co-IP and western blot. **E** Co-IP and western blot assessed the interaction of DCDC2, ENO1, FBXW7, and NEDD4L. ***p < 0.001

Moreover, DCDC2 overexpression increased the protein levels of ENO1, whereas DCDC2 knockdown reduced ENO1 protein levels in ICC cells (Fig. 6A). Protein degradation experiments showed that DCDC2 overexpression led to a longer half-life of ENO1 protein in Cycloheximide (CHX) -treated ICC cells (Fig. 6B). Autophagy and the ubiquitin–proteasome system are the two principal protein degradation pathways [26]. Chloroquine (CQ) and MG132 were introduced to inhibit autophagy and ubiquitination degradation in ICC cells, respectively. MG132 treatment could partially rescue the decrement of ENO1 protein in DCDC2 knockdown cells (Fig. 6C), while CQ treatment could not, suggesting that DCDC2 could stabilize ENO1 protein through regulating ubiquitin–proteasome pathway. Indeed, DCDC2 overexpression decreased ENO1 ubiquitination (Fig. 6D). It has been reported that FBXW7 and NEDD4L are the E3 ligases that regulate ENO1 ubiquitination [27, 28]. The binding capacity of FBXW7 and NEDD4L with ENO1 was further evaluated under DCDC2 overexpression. The results showed that FBXW7 interacts with ENO1 and the association was dampened in the DCDC2-overexpressed ICC cells (Fig. 6E and Supplementary Fig. 4L). By regulating the ubiquitination-mediated degradation of key oncogenic proteins, FBXW7 plays a crucial role in the development and progression of tumors [29, 30]. Several studies have reported that FBXW7 is involved in the ubiquitination-mediated degradation of ENO1 [31–35]. In ICC, we observed that following the overexpression of DCDC2, the binding affinity of FBXW7 to ENO1 is weakened, and ENO1 ubiquitination was decreased. These results imply the involvement of FBXW7 in DCDC2-related suppression of ENO1 ubiquitination.

ENO1 is a moonlighting protein that performs multiple biochemical functions [36], and is a critical regulator of AKT phosphorylation in several types of cancers [37, 38]. ENO1 was knocked down in ICC wild-type cell lines, and knockdown of ENO1 significantly reduced the phosphorylation of AKT (Supplementary Fig. 5 A). Further rescue assays were designed to validate the role of ENO1 in the DCDC2-AKT axis. ENO1 knockout diminished the enhancement of AKT phosphorylation which was induced by DCDC2 (Supplementary Fig. 5B). Colony formation assays and CCK8 assays confirmed that ENO1 knockout decreased the DCDC2-induced enhancement of ICC cell proliferation (Supplementary Fig. 5 C and 5D). Transwell analysis also demonstrated that depletion of ENO1 dampened the migration and invasion abilities of DCDC2-overexpressed ICC cells (Supplementary Fig. 5E and 5 F). Collectively, these results demonstrated that ENO1 is a functional downstream effector which was responsible for DCDC2-mediated oncogenic functions in ICC cells.

### ENO1 promotes FGL1 expression by binding to FGL1 promoter

**Fig. 7:**
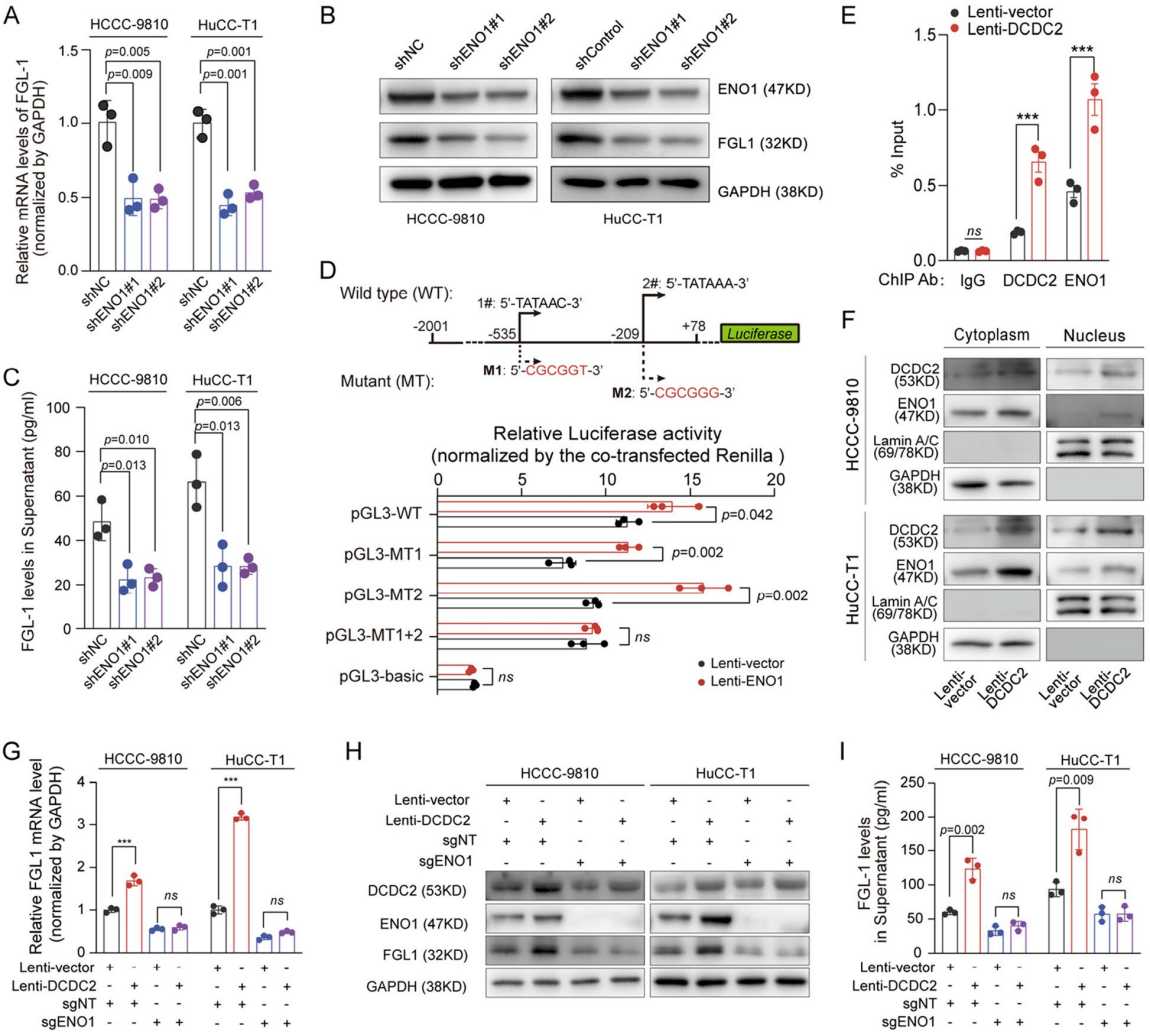
ENO1 promotes the transcription of FGL1. **A** The relative expressions of FGL1 mRNA in ICC cells were assessed by qPCR after ENO1 knockdown. **B** The protein levels of ENO1 were assessed by western blot after ENO1 knockdown. **C** The protein levels of FGL1 in supernatant of ICC were assessed by ELISA after ENO1 knockdown. **D** The two predicted binding sites and the corresponding mutants of ENO1 in FGL1 promoter region and relative luciferase activity in dual-luciferase reporter assay. **E** ENO1- or DCDC2-associated chromatin complexes were immunoprecipitated from HuCC-T1-Vector and HuCC-T1-DCDC2-OE cells. Precipitated DNA was analyzed for FGL1 promoter enrichment by qPCR (DCDC2-OE vs. Vector; IgG control). **F** The expressions of DCDC2 and ENO1 in cytoplasm and nucleus of ICC cells after DCDC2 overexpression were assessed by WB. **G** The relative expressions of FGL1 mRNA in DCDC2 overexpressed and ENO1 knocked-out cells were assessed by qPCR. **H** The protein levels of FGL1 in DCDC2 overexpressed and ENO1 knocked-out cells were assessed by western blot. **I** The protein levels of FGL1 in supernatant of DCDC2 overexpressed and ENO1 knocked-out cells were assessed by ELISA. ***p < 0.001

We found that DCDC2 interacts with and stabilizes ENO1, which in turn activates AKT to promote ICC progression. In addition to the oncogenic effects on ICC cell per se, DCDC2 has been above proved that could promote ICC immune evasion through upregulating FGL1-related immune checkpoint. ENO1, the chief protein partner of DCDC2 in ICC cells, has been reported to act as a DNA-binding protein to regulate target gene transcription [39]. We therefore speculate that DCDC2 might regulate the expression of FGL1 through ENO1. Not surprisingly, ENO1 knockdown significantly reduced the mRNA level of FGL1 in mRNA levels (Fig. 7A), together with unquestioned decreased intracellular protein level (Fig. 7B) and protein level in supernatant (Fig. 7C). There are two predicted ENO1 binding sites embedded in FGL1 promoter (the upper of Fig. 7D**,** JASPER: http://jaspar.binf.ku.dk). Mutant sequences were introduced into these two sites, and luciferase reporter assay showed that the regulatory effects of ENO1 on the FGL1 promoter were erased when both of them were mutated (the bottom of Fig. 7D). ChIP-qPCR analysis further confirmed that DCDC2 and ENO1 co-occupy the FGL1 promoter as a complex (Fig. 7E). The distribution of ENO1 were examined, and it turned out that DCDC2 could upregulate the protein level of ENO1 both in the cytoplasm and the nucleus of ICC cells (Fig. 7F and Supplementary Fig. 6 A). Moreover, ENO1 knockdown eliminated the DCDC2-promoting effects on FGL1 expression, resulting in that the mRNA level, intracellular protein level and supernatant protein level of FGL1 were all remarkably decimated (Fig. 7G-I). Further, we assessed whether Akt activation is involved in the regulation of FGL1. The results showed that perifosine treatment did not influence the expression of FGL1 (Supplementary Fig. 6B). Together, these data demonstrated that ENO1 promotes the transcriptional regulation of FGL1, which is responsible for DCDC2-mediated ICC immune evasion.

### LAG-3 antibody treatment inhibited the reduction of CD8^+^ T cell function induced by DCDC2 overexpression

**Fig. 8:**
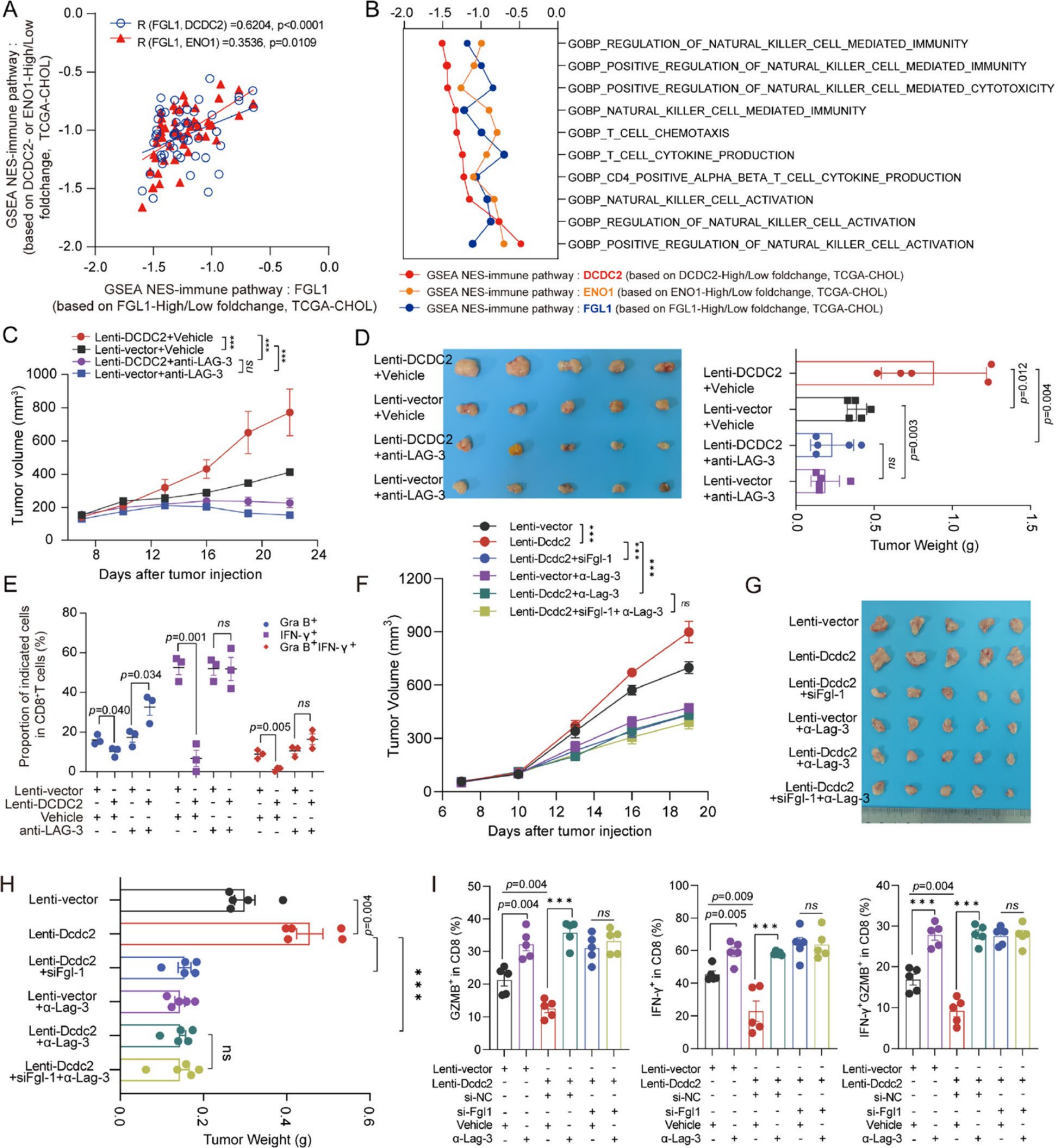
FGL-LAG-3 checkpoint is involved in DCDC2-induced immune evasion. **A** Correlation analysis was conducted on the immune signaling pathways enriched by GSEA in the high DCDC2 expression group with the immune signaling pathways enriched by the high expression groups of FGL1 or ENO1 in the TCGA-CHOL databases. **B** The TCGA-CHOL dataset was divided into high and low expression groups based on the median values of DCDC2/ENO1/FGL1 expression. Genes that were differentially expressed within the high DCDC2/ENO1/FGL1 expression group compared to the low DCDC2/ENO1/FGL1 group were subjected to GSEA for immune-related pathways.** C** Tumor growth curves of humanized mice inoculated with the vehicle or DCDC2 overexpressed HuCC-T1 cells treated with or without α-LAG-3. **D** Image (left) and tumor weight (right) of subcutaneous xenograft tumor of humanized mice inoculated with the vehicle or DCDC2 overexpressed HuCC-T1 cells treated with or without α-LAG-3. **E** The expressions of granzyme B and IFN-γ in CD8^+^ T cells in xenograft tumors of humanized mice were assessed by flow cytometry. **F** Tumor growth curves of subcutaneous tumors of syngeneic model. **G&H** Image (left) and tumor weight (right) of subcutaneous tumors of syngeneic model. **I** The expressions of granzyme B and IFN-γ in CD8^+^ T cells in subcutaneous tumors of syngeneic model were assessed by flow cytometry. ***p < 0.001

In line with the regulatory effects of DCDC2-ENO1 axis on FGL1 expression, further GSEA analysis was carried out on the dataset of TCGA-CHOL classified by DCDC2, ENO1 or FGL1 expression (High or Low by Mean value). The immuno-potentiating pathways were mostly negatively regulated by each of these genes, and the regulatory effects of DCDC2 or FGL1 on immune pathways were in a closely positive correlation (Fig. 8A). Similar results were also found in the regulatory effects of ENO1 or FGL1 on immune pathways, indicating the existence of the long-acting regulatory DCDC2-ENO1-FGL1 axis. Consistently, the biological pathways involved in natural killer cell activation, T cell activation and the related cytokine production were also negatively regulated by each of these genes (Fig. 8B). The in vitro* and *in vivo assays were designed to determine whether FGL1/LAG-3 immune checkpoint takes part in the immune evasion induced by DCDC2 in ICC. The in vitro co-culture model of SB-1 cell and PBMCs was additionally treated with the Lag-3 antibodies and the function of CD8^+^ T cells was detected. The treatment of Lag-3 antibodies suppressed the downregulation of Granzyme B, IFN-γ, and Ki-67 in CD8^+^ T cells induced by overexpression of Dcdc2 (Supplementary Fig. 7). The humanized mouse xenograft models were treated with LAG-3 antibodies. The engraftment levels of hCD45^+^ cells in humanized mice were determined 2 weeks post-PBMCs transplantation by flow cytometry (Supplementary Fig. 8 A). The results showed that LAG-3 antibody treatment abolished the enhancement of tumor growth induced by DCDC2 overexpression in humanized mice (Fig. 8C and D), both in the tumor volume and the tumor weight. Moreover, the expression of Granzyme B and IFN-γ in CD8^+^ T cells in the humanized mouse spleen was examined. The results showed that treatment of LAG-3 antibodies recovered the downregulation of Granzyme B and IFN-γ in CD8^+^ T cells induced by overexpression of DCDC2 (Supplementary Fig. 8B and 8 C). Similar results were observed in the humanized mouse xenograft tumor (Fig. 8E and Supplementary Fig. 8D).

**Fig. 9:**
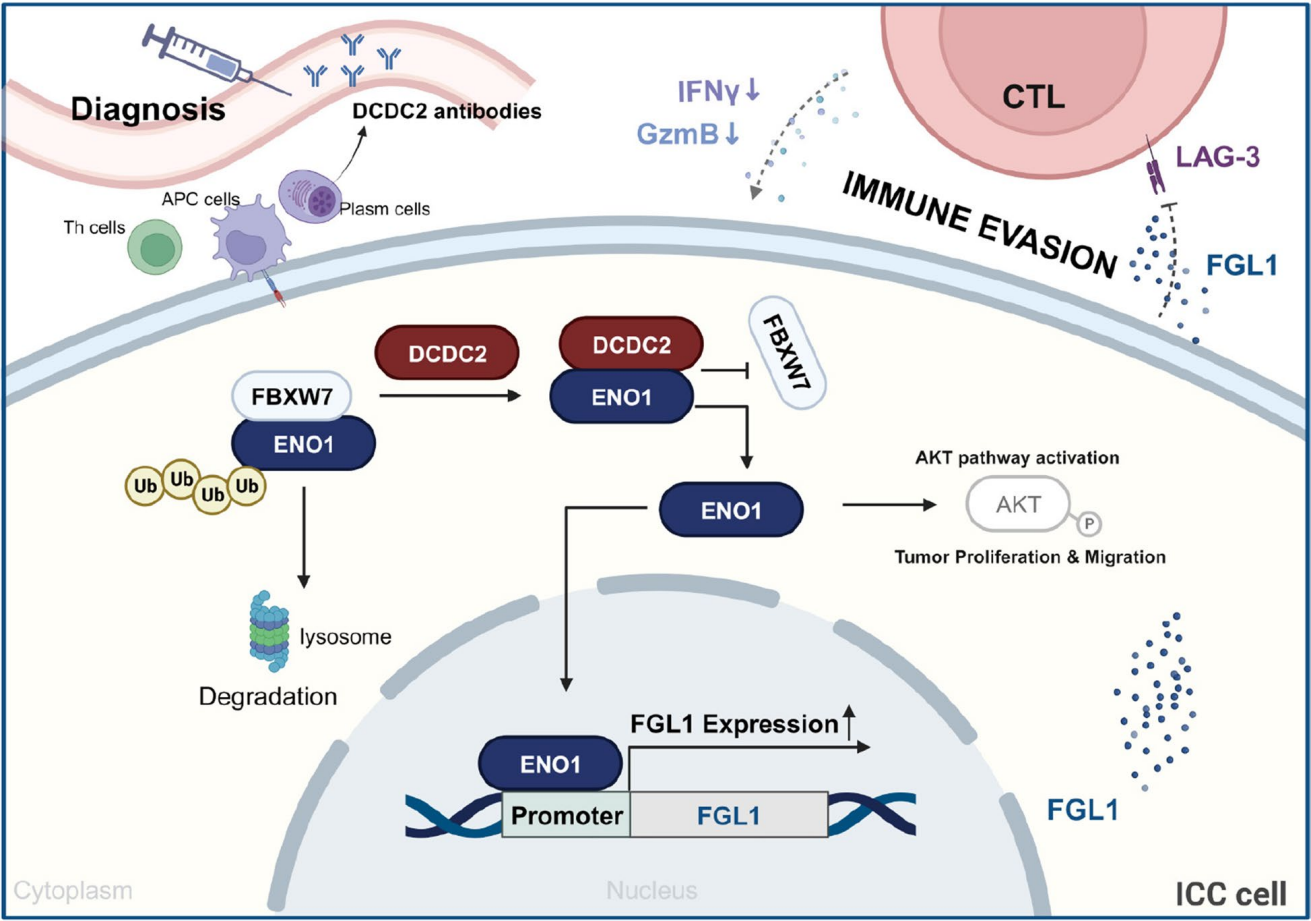
Diagram of the DCDC2 role in ICC

We next verified the function of DCDC2-FGL1-LAG-3 axis in the syngeneic model. In vivo assays revealed that treatment with Lag-3 antibodies negated the tumor growth enhancement associated with Dcdc2 overexpression, while Fgl1 knockdown blocked the efficacy of Lag-3 antibodies (Fig. 8F, G, H and Supplementary Fig. 8E). Additionally, we investigated the expression levels of Granzyme B and IFN-γ in CD8^+^ T cells within the tumors, confirming similar trends (Fig. 8I and Supplementary Fig. 8 F). Similar results were observed in co-culture model of SB-1 cell and PBMCs (Supplementary Fig. 7). These results supported that the DCDC2-ENO1-FGL1/LAG-3 axis promotes immune evasion of ICC cells. In summary, DCDC2 is identified as an ICC-associated antigen that is abundantly expressed in ICC. DCDC2 interacts with and stabilizes ENO1, which in turn enhances AKT phosphorylation and promotes ICC progression. Additionally, ENO1 binds to the FGL1 promoter to upregulate FLG1 expression, thereby inhibiting CD8^+^ T cell function through the FGL1-LAG3 axis (Fig. 9).

## Discussion

In tumor patients, the immune system may recognize TAAs in the early stages of tumor development and produce corresponding TAAbs. This biological amplification process allows for the detection of these antibodies before the tumor antigens themselves have been discovered. Meanwhile, the TAAbs in the peripheral blood of patients are much more abundant than TAAs and have a longer half-life. Therefore, TAAbs could be useful biomarkers for early cancer diagnosis [12, 40]. Several studies had revealed the value of TAAbs in cancer diagnosis. Autoantibodies to two TAAs, p62 and Koc, have been detected in various malignancies, showing a significant difference from the control populations [41]. The combination of different TAAbs showed promising diagnostic efficiency in lung cancer and colon cancer [42, 43]. In this study, we found that the level of anti-DCDC2 autoantibody in serum of patients with ICC is elevated, suggesting that it is a potential biomarker for early diagnosis of ICC. Moreover, we found that in the normal bile duct, the DCDC2 protein was enriched in the cell membrane inside the bile duct. However, in ICC tissue, it exhibited disorganized distribution and aberrant localization. This might partially explain the production of anti-DCDC2 autoantibodies in ICC patients [13, 44].

Besides, some TAAs have important cellular biological functions in cancer cells. For example, the cell cycle protein Cyclin B1, which plays a key role in the progression of the cell cycle, has been identified as TAA in several cancers [45, 46]. Antibodies to p53, a classical tumor suppressor protein that influences various aspects of tumorigenesis and biological behavior, have been found in patients with lung cancer and breast cancer [47, 48]. We then explored the function of the DCDC2 protein in ICC. DCDC2 is a microtubule-associated protein that participate in neuronal cell migration and primary cilia formation [16, 19, 22]. However, few studies have explored the function of DCDC2 in cancer. Longoni et al. [49] found that the aberrant expression of DCDC2 promotes malignant phenotypes in prostate cancer. Recently, it was discovered that DCDC2 plays a role in the progression of colorectal cancer by interacting with DVL2 to regulate the Wnt pathway [50]. In the current study, we found that DCDC2 could promote the proliferation and metastasis of ICC by regulating AKT phosphorylation. DCDC2 can also upregulate FGL1 to promote ICC immune evasion. Considering the specifically high expression and abnormal localization of DCDC2, it may be a promising therapeutic target for ICC.

ENO1 is one of the enolases expressed in endothelial cells. It had been reported that CCDC65 can interact with the TIM barrel domain of ENO1 and promoted ubiquitylation of ENO1 by recruiting FBXW7 [31]. The CCDC65 protein participates in the assembly of the nexin-dynein regulatory complex, and mutations in CCDC65 gene can result in primary ciliary dysfunctions [51, 52]. In our study, we found that DCDC2, another microtubule-associated protein and primary cilia-associated protein, can also interact with the TIM barrel domain of ENO1 through its functional DCX domain and reduced the ubiquitination of ENO1 by FBXW7. These results indicate a correlation between ENO1 and primary cilia-associated proteins.

ENO1 has been identified as a moonlight protein that plays critical noncanonical functions of metabolic enzymes in cancer [36]. ENO1 can promote tumor progression by interacting with and promoting AKT phosphorylation [37]. In this study, we found that DCDC2 regulates AKT phosphorylation, thereby promoting proliferation, migration, and invasion in ICC cells by stabilizing ENO1. The serine/threonine kinase AKT is involved in the regulation of multiple signaling pathways [53]. In biliary tract cancer, AKT gene mutation and copy number variation are infrequent [54, 55]. However, overexpression and activation of AKT are common. IHC-positive AKT and its phosphorylated form (p-AKT) were found in 46–100% and 34–100% of biliary tract cancer tissue samples [56]. The activation of the AKT pathway in ICC has been reported to be mediated by upstream proteins such as MUC13 and PLCB1 [57, 58]. Our results, demonstrating that DCDC2 promotes AKT phosphorylation by stabilizing ENO1, reveal a novel regulatory mechanism of AKT in ICC.

Furthermore, ENO1 can also act as a DNA or RNA binding protein to regulate transcription and translation in cancer cells [59]. For example, ENO1 can bind to c-myc promoter and regulates the expression of c-myc [39]. FGL1 protein is primarily secreted by hepatocytes and contributes to mitogenic and metabolic functions under normal physiological conditions [60]. Recent years, FGL1 was found to be overexpressed in tumors. FGL1 expression is regulated by inflammation factors such as interleukin 6 and transforming growth factor TGF-β [61, 62]. Our data verified a new mechanism of FGL1 regulation that ENO1 can bind to the two binding sites to promote FGL1 expression. Our results demonstrated that DCDC2 could enhance the accumulation of ENO1 protein both in the cytoplasm and the nucleus of ICC cells. It has been acknowledged that de-ubiquitylation could modulate the nuclear translocation of particular proteins [63, 64]. Considering the DCDC2-related suppression of ENO1 ubiquitination in ICC cells, we speculate that DCDC2 overexpression may also promote ENO1 nuclear translocation. However, further studies are needed to validate this hypothesis.

The GO enrichment analysis in our results indicated that the natural killer (NK) cell and T cell-associated signaling pathways were enriched. In the allograft tumor-bearing model, we observed that the function of NK cells was decreased in the Dcdc2 overexpression group (data not shown). We strategically focused on T cell biology based on three key considerations. First, CD8^+^ T cells serve as the central effectors of adaptive antitumor immunity through MHC-I-dependent antigen recognition, whereas NK cell activity is largely confined to"missing-self"targeting of MHC-I-deficient cells—a mechanism less prevalent in immunoedited tumors [65, 66]. Second, existing clinically validated immunotherapies (e.g., anti-PD-1/CTLA-4) specifically target T cell checkpoints, making mechanistic insights into T cell regulation by DCDC2 directly translatable to combination strategies with approved regimens. Finally, emerging evidence indicates that reinvigorated T cells can enhance NK cell recruitment and function via cytokine networks (e.g., IFN-γ/CCL5) [67, 68], suggesting that targeting T cell dysfunction may synergistically restore both adaptive and innate immunity. Therefore, we believe that research on T cells can leverage existing mature drugs to address clinical issues, thereby benefiting patients. Chen et al. [25] demonstrated that FGL1 is a major inhibitory ligand of LAG-3, which can suppress antitumor T-cell responses. Our results are consistent with the previous study that FGL1, serving as ligand of LAG-3, inhibits the function of CD8^+^ T cells and promotes immune evasion of ICC. There is increasing evidence that the intercellular communication in the tumor microenvironment of ICC creates an immunosuppressive microenvironment in most patients [9]. The present study uncovered a DCDC2/ENO1/FGL1/LAG-3 axis and communication between ICC tumor cells and CD8^+^ T cells. Our results showed the potential therapeutic value of targeting the axis.

There are some limitations in our study. Firstly, without a normalized curve of anti-DCDC2 autoantibody titer, we were unable to propose a cut-off value of the antibody for ICC diagnosing. Secondly, further study is needed to determine whether anti-DCDC2 autoantibodies would influence the progression of ICC. Thirdly, while external validation is essential, ICC's rarity restricted cohort expansion during this study. Moving forward, we are addressing this through ongoing multicenter collaborations to standardize cut-offs and validate findings across populations.

## Conclusion

In summary, our study reveals that DCDC2 is a prominent ICC-associated antigen, highly expressed in ICC and correlated with poor prognosis. The presence of anti-DCDC2 autoantibodies may serve as a diagnostic biomarker for ICC. Furthermore, DCDC2 facilitates the progression and immune evasion of ICC. Mechanistically, DCDC2 interacts with and stabilizes ENO1, which in turn enhances AKT phosphorylation and binds to the FGL1 promoter, leading to increased FGL1 expression. Notably, elevated FGL1 levels significantly impair CD8^+^ T cell functionality through the FGL1-LAG3 axis. These findings introduce a novel diagnostic marker and therapeutic target for ICC patients.

## Impact and implications

Intrahepatic cholangiocarcinoma (ICC) is a malignant tumor with insidious symptoms and poor prognosis, highlighting the urgent need for early diagnostic methods and effective therapies. Our study identifies DCDC2 as a promising diagnostic biomarker for ICC, with elevated anti-DCDC2 autoantibodies indicating potential for early detection. DCDC2 promotes ICC proliferation, metastasis, and immune evasion by stabilizing ENO1, enhancing AKT phosphorylation, and increasing FGL1 levels, which impairs CD8^+^ T cell functionality. These findings suggest new avenues for targeted therapies to improve patient outcomes.

## Supplementary Information

Supplementary Material 1

## Data Availability

All data pertinent to this study, whether generated or analyzed, are comprehensively presented in this manuscript and its supplementary information. For any additional inquiries or requests, interested parties are encouraged to contact the corresponding authors.

## Abbreviations

CCA: Cholangiocarcinoma
ICC: Intrahepatic Cholangiocarcinoma
PBMC: Peripheral blood mononuclear cell
ELISA: Enzyme linked immunosorbent assay
DCDC2: Doublecortin domain containing 2
FGL1: Fibrinogen-like protein 1
ENO1: Enolase 1
PHCC: Perihilar duct cholangiocarcinoma
DCC: Distal duct cholangiocarcinoma
ECC: Extrahepatic cholangiocarcinoma
OS: Overall survival
ICIs: Immune checkpoint inhibitors
TAAs: Tumor-associated antigens
DCX: Doublecortin
TILs: Tumor-infiltrating lymphocytes
MS: Co-IP/Mass Spectrometry
P/S: Penicillin-streptomycin
CQ: Chloroquine
scRNA-seq: Single-cell RNA sequencing
UMAP: Uniform manifold approximation and projection
NK cell: Natural killer cell
TAMs: Tumor-associated macrophages
CAFs: Cancer-associated fibroblasts
TECs: Tumor endothelial cells
LAG-3: Lymphocyte activation gene 3
q-PCR: Quantitative real time polymerase chain reaction
IHC: Immunohistochemistry
TCGA: The Cancer Genome Atlas
