# Supplementary material for: The DCDC2/ENO1 axis promotes tumor progression and immune evasion in intrahepatic cholangiocarcinoma via activating FGL1-LAG3 checkpoint
Source: J Exp Clin Cancer Res. 2025 Jun 18;44:177. doi: 10.1186/s13046-025-03436-1 (PMC12175362; doi:10.1186/s13046-025-03436-1)
Supplement: Supplementary file 1 — Supplementary Material 1 [file 13046_2025_3436_MOESM1_ESM.doc]

**The DCDC2/ENO1 axis promotes tumor progression and immune evasion in intrahepatic cholangiocarcinoma via activating FGL1-LAG3 checkpoint**

**Wenze Wan**^1,2,3,†^, **Yuan Li**^2,†^, **Wentao Sun**^1,4,5,†^, Zewei Cheng^2^, Fen Ma^2^, Sheng Shen ^1,4,5,*^, Houbao Liu^1,4,5,*^, and Jiwei Zhang^2,6,*^

1. Department of Biliary Surgery, Zhongshan Hospital, Fudan University, Shanghai 200032, China.
2. Shanghai Key Laboratory of Compound Chinese Medicines, The MOE Key Laboratory for Standardization of Chinese Medicines, Institute of Chinese Materia Medica, Shanghai University of Traditional Chinese Medicine, Shanghai, 201203, China.
3. Department of Gastrointestinal Surgery, Zhongnan Hospital of Wuhan University, Wuhan, 430000, China.
4. Biliary Tract Disease Institute, Fudan University, Shanghai, 200032, China.
5. Shanghai Biliary Tract Minimal Invasive Surgery and Materials Engineering Research Center, Shanghai, 200032, China.
6. Lead contact

**Running title:** DCDC2 promotes progression and immune evasion of ICC

† These authors contributed equally.

* Correspondence

Jiwei Zhang, Shanghai Key Laboratory of Compound Chinese Medicines, Institute of Chinese Materia Medica, Shanghai University of Traditional Chinese Medicine, 5325 Rm, 5# Bldg. 1200 Cai Lun Road, Shanghai 201203, China,

Email: joezhang@shutcm.edu.cn.

Houbao Liu, Department of Biliary Surgery, Zhongshan Hospital, Fudan University, Shanghai 200032, China,

Email: zsliuhb@sina.com.

Sheng Shen, Department of Biliary Surgery, Zhongshan Hospital, Fudan University, Shanghai 200032, China,

Email: shen.sheng@zs-hospital.sh.cn.

**Supplementary Figure**

**Supplementary Figure 1**

**
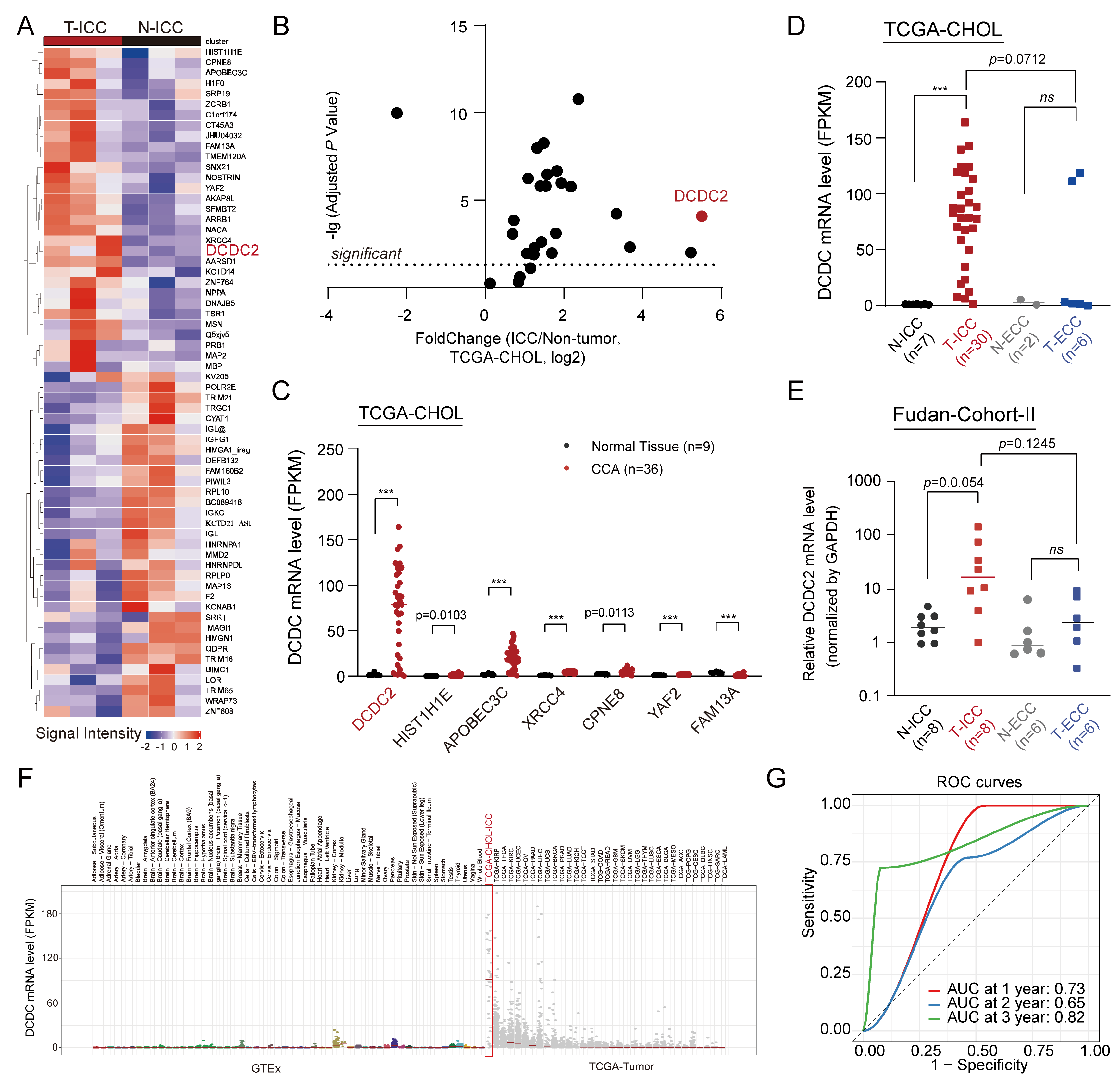
**

**Supplementary Figure 1. DCDC2 expression in public database. A** Heat map of differential autoantibodies in healthy donors and CCA patients. **B** Volcano plot of TAAbs corresponding genes in the TCGA-CHOL cohort. **C** Expression of the 7 differentially expressed genes in the TCGA-CHOL cohort. **D** Expression of DCDC2 of ICC and ECC in the TCGA-CHOL cohort. **E** The Expressions of DCDC2 in Fudan-Cohort-II. **F** Expression of DCDC2 in the GTEx database and TCGA database. G. The ROC curves for Prognostic Stratification of DCDC2 protein. ****p* < 0.001.

**Supplementary Figure 2**


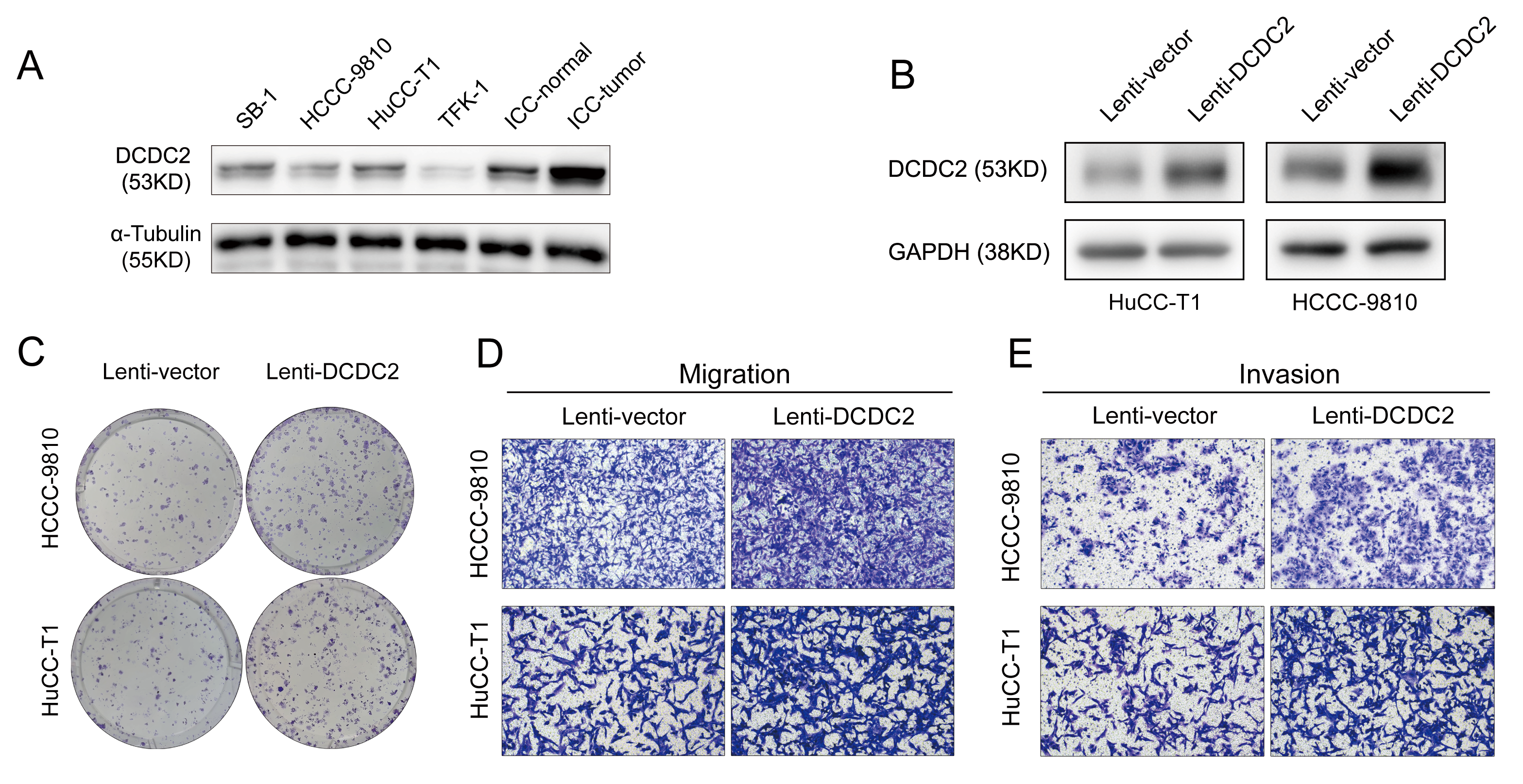


**Supplementary Figure 2.** **ENO1 mediates the effect of DCDC2 on proliferation, migration, and invasion. A** Western blot was performed to assess the expression of DCDC2 in SB-1, HCCC-9810, HuCC-T1, TFK-1 cell lines and ICC tissues. **B** DCDC2 overexpression in ICC cells. **C** The representative image of Colony formation assays. **D** The representative image of Transwell migration assays. **E** The representative image of Transwell Invasion assays.

**Supplementary Figure 3**


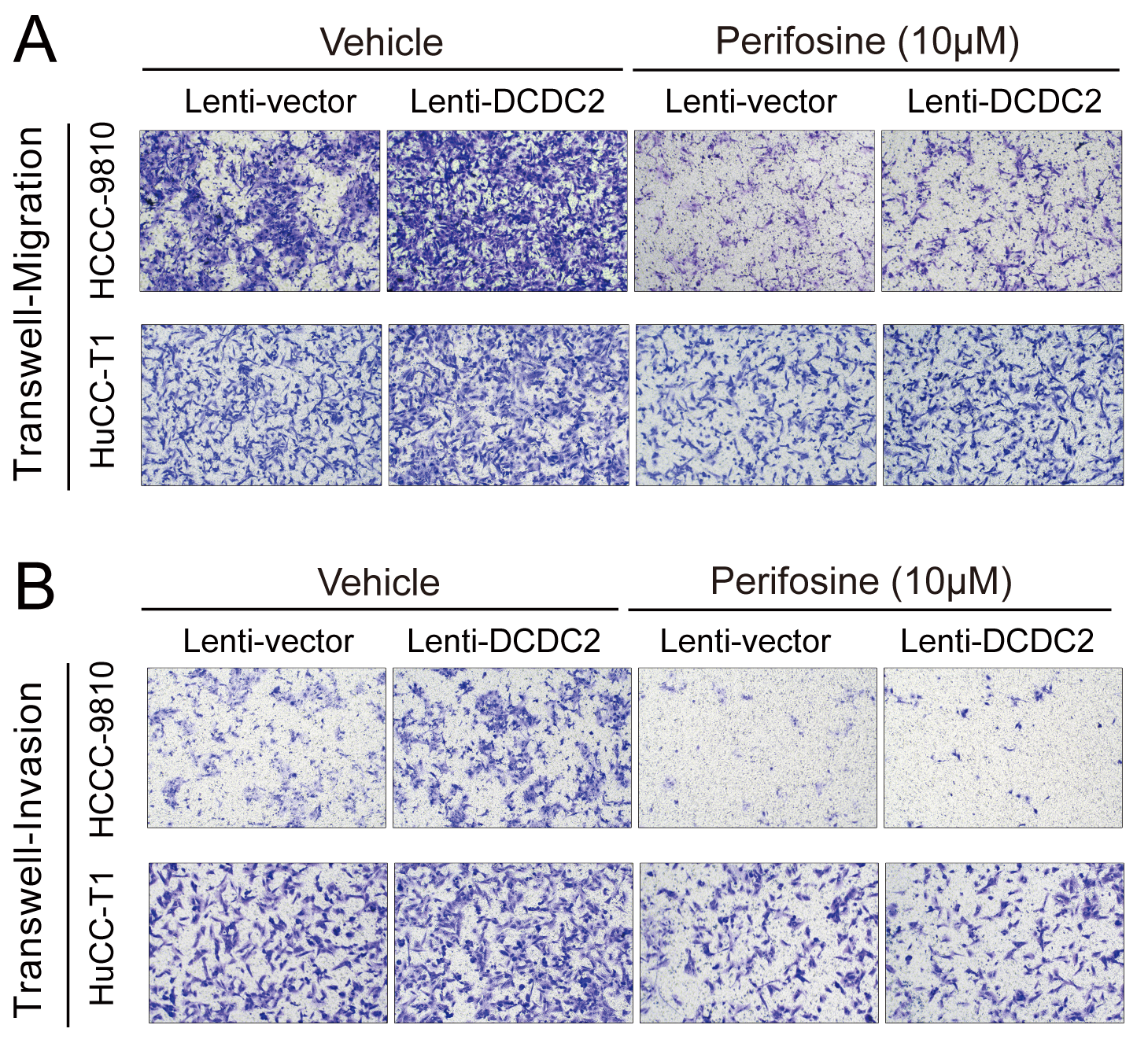


**Supplementary Figure 3.** **The effect of Perifosine on migration and invasion of DCDC2 overexpressed cells. A** Representative image of transwell migration assay in DCDC2 overexpressed cells treated with Perifosine(10μM). **B** Representative image of transwell invasion assay in DCDC2 overexpressed cells treated with Perifosine (10μM).

**Supplementary Figure 4**


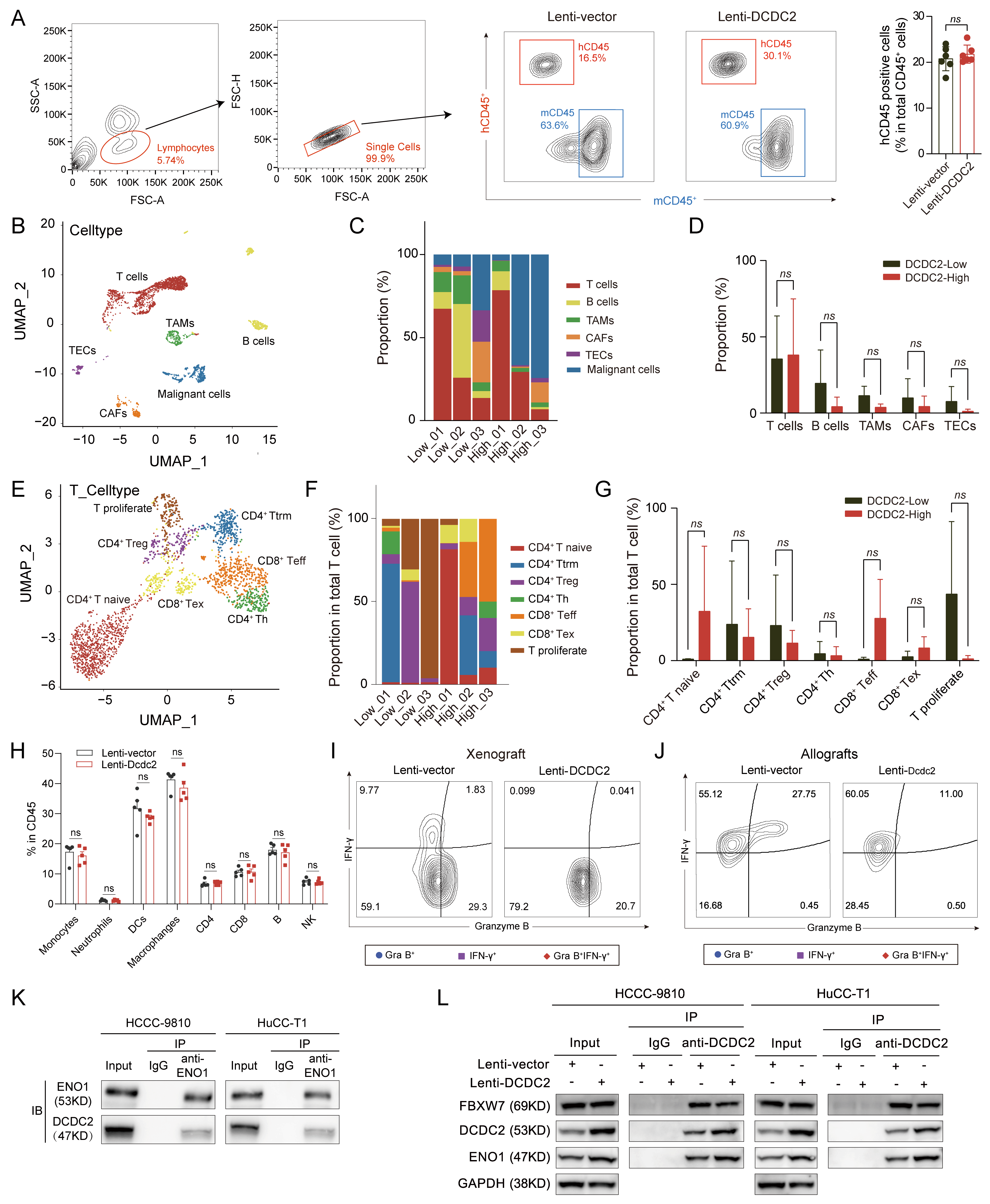


**Supplement Figure 4. A** The engraftment levels of hCD45^+^ cells were determined 2 weeks post-PBMCs transplantation by flow cytometric. **P* < 0.05, ***P* < 0.01, ****P* < 0.001. **B** UMAP plot of the six cell types. **C** Proportions of the six cell types in the six samples. **D** Comparison of the proportion of cell types between DCDC2-high and DCDC2-low group. **E** UMAP plot of T cell subsets. **F** Proportions of T cell subsets in the six samples. **G** Comparison of the proportion of T cell subsets between DCDC2-high and DCDC2-low group. **H** The proportion of immune cells in tumors of the syngeneic model. **I** The expressions of granzyme B and IFN-γ in CD8^+^ T cells in xenograft tumors of humanized mice were assessed by flow cytometry. **J** The expressions of granzyme B and IFN-γ in CD8^+^ T cells in allograft tumor of syngeneic model were assessed by flow cytometry. K Co-IP and Western blot verified the interaction of DCDC2 and ENO1. L Co-IP and Western blot verified the interaction of DCDC2, ENO1, and FBXW7. ****p* < 0.001.

**Supplementary Figure 5**

**
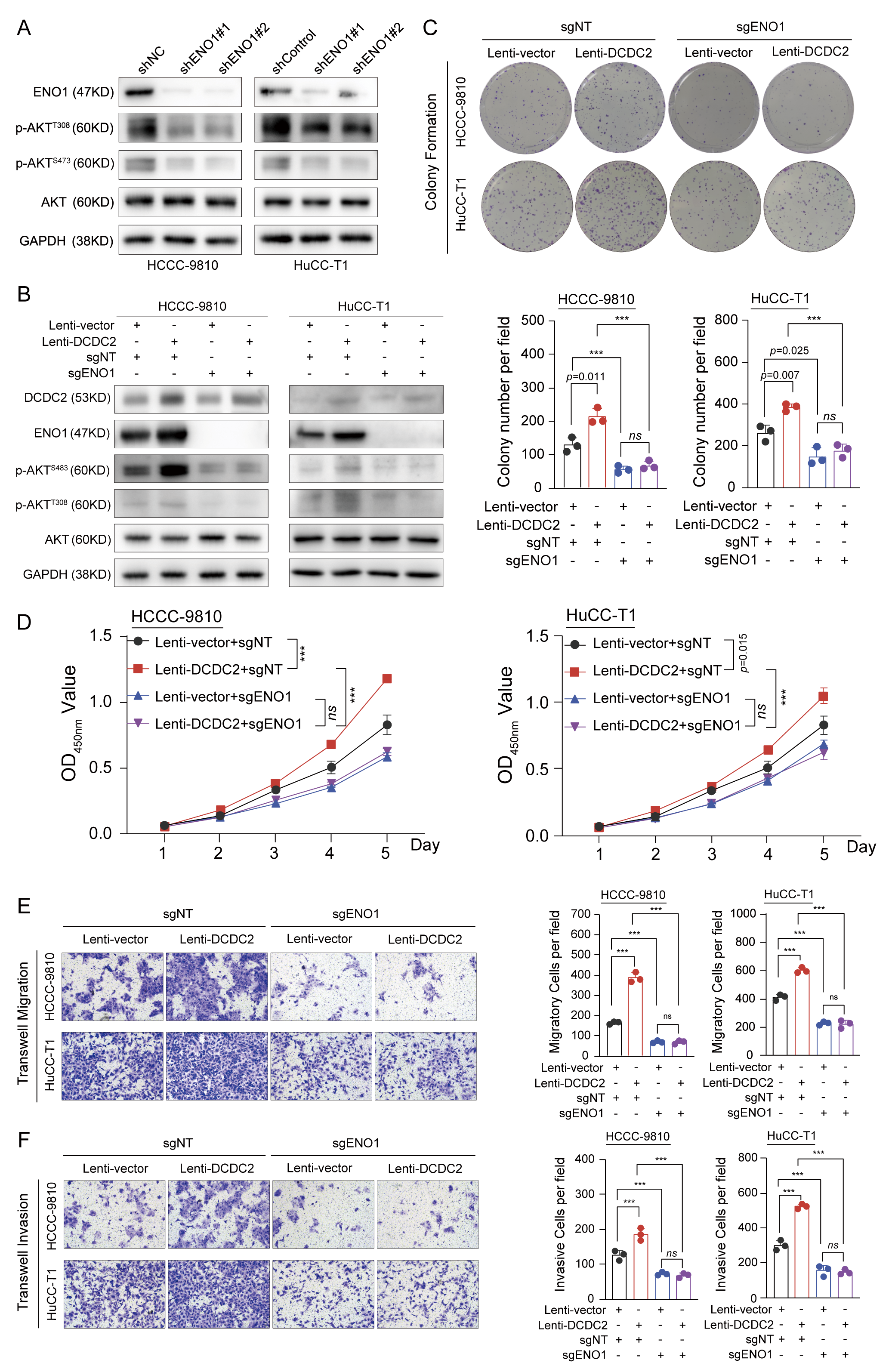
**

**Supplement Figure 5. ENO1 mediates the effect of DCDC2 on proliferation, migration, and invasion. A** The protein levels of p-AKT^T308^, p-AKT^S473^, and AKT were assessed by western blotting after ENO1 knockdown. **B** The protein levels of p-AKTT308, p-AKTS473, and AKT were assessed by western blotting after DCDC2 overexpression and ENO1 knockout. Colony formation assays (**C**) and CCK8 assays (**D**) showed knockout of ENO1 decreased the enhancement of ICC cell proliferation induced by DCDC2 overexpression. **E** Transwell migration assay showed depletion of ENO1 weakened the migration ability in DCDC2 overexpression cell lines. **F** Transwell invasion assay showed depletion of ENO1 weakened the invasion ability in DCDC2 overexpression cell lines. ****p* < 0.001.

**Supplementary Figure 6**

**
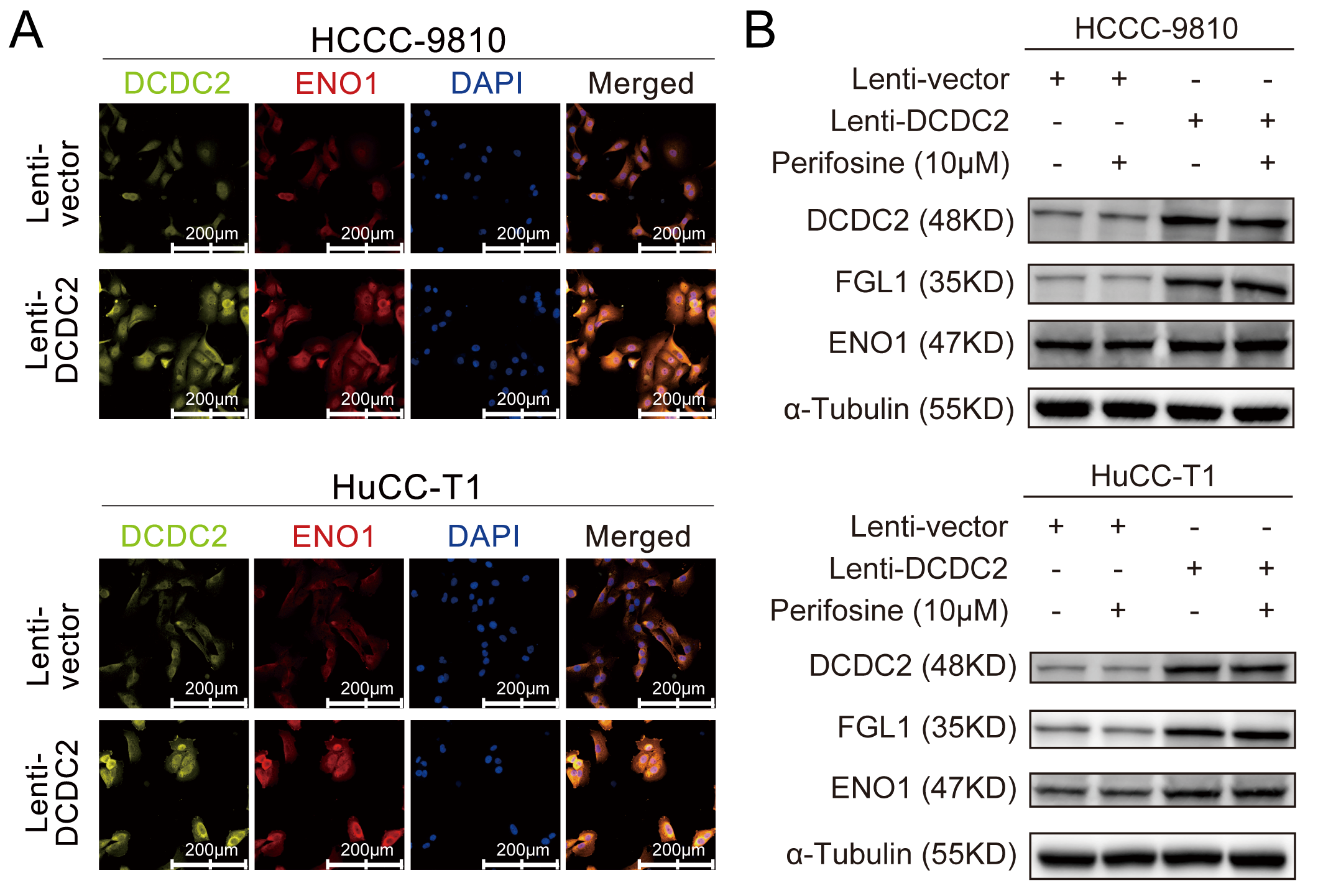
**

**Supplement Figure 6. A** Representative image of Immunofluorescence DCDC2 and ENO1 in ICC cells. **B** The protein levels of DCDC2, ENO1, FGL1 were assessed by western blotting after perifosine treatment.

**Supplementary Figure 7**

**
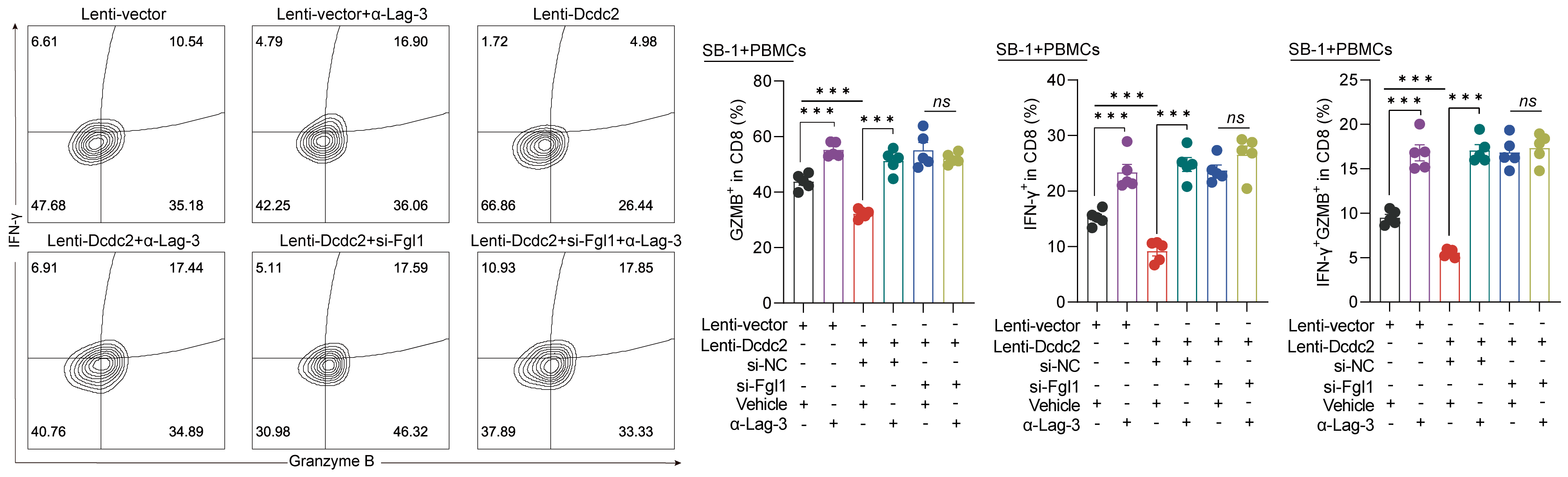
**

**Supplementary Figure 7** The expressions of granzyme B and IFN-γ in CD8^+^ T cells in co-culture model of SB-1 cell and PBMCs were assessed by flow cytometry.

**Supplementary Figure 8**

**
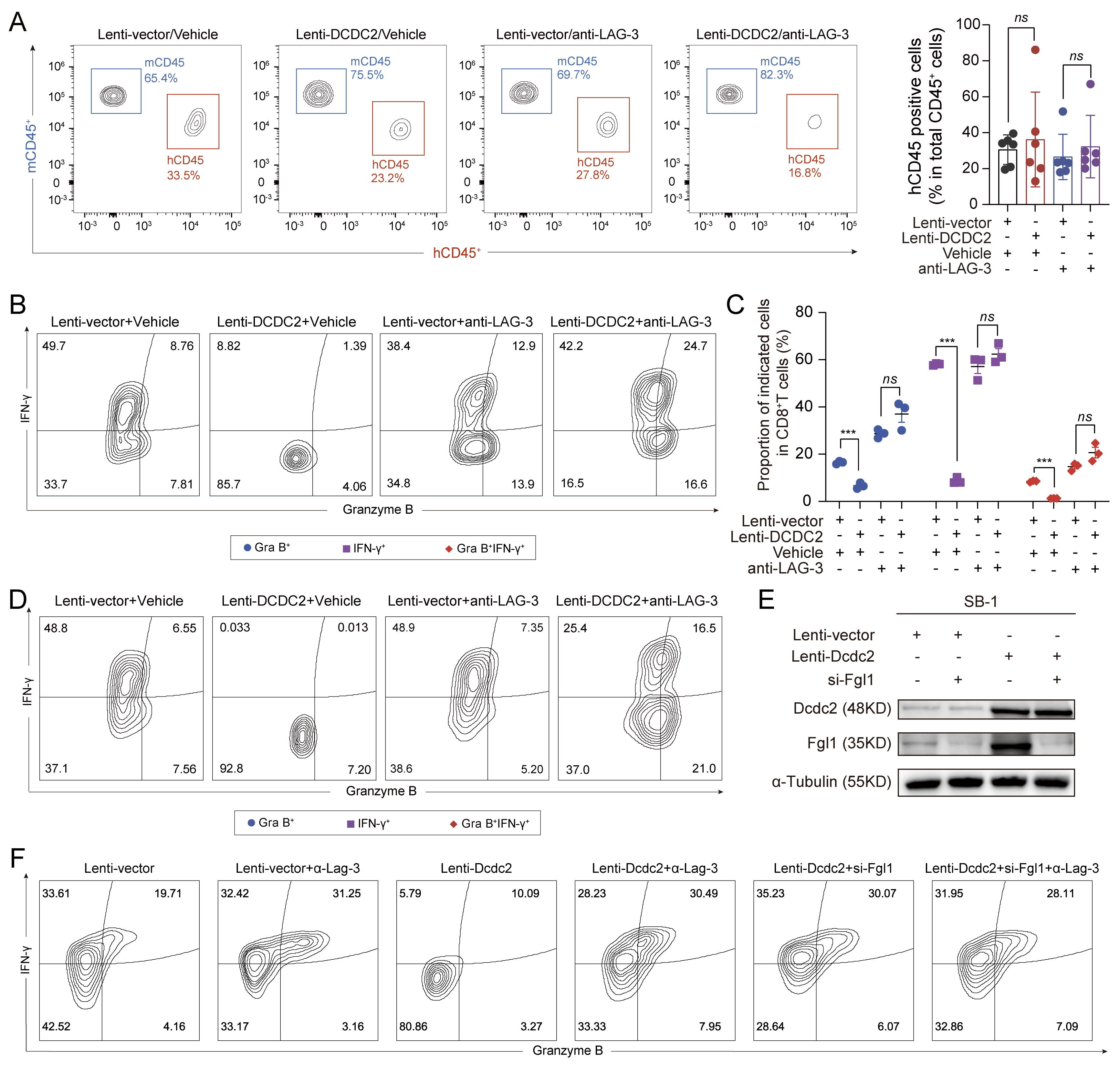
**

**Supplement Figure 8 A** The engraftment levels of hCD45^+^ cells were determined 2 weeks post-PBMCs transplantation by flow cytometric in humanized mice xenograft models treated with or without α-LAG-3. **B&C** The expressions of granzyme B and IFN-γ in CD8^+^ T cells in spleens of humanized mice were assessed by flow cytometry. **D** Representative Image of the expressions of granzyme B and IFN-γ in CD8^+^ T cells in xenograft tumors of humanized mice. **E** The protein levels of Dcdc2, Fgl1 were assessed by western blotting after Dcdc2 overexpression or Fgl1 knock-down. **F** Representative Image of the expressions of granzyme B and IFN-γ in CD8^+^ T cells in subcutaneous tumors of syngeneic model. ****p* < 0.001.

**Supplementary Table**

**Supplementary Table 1. FUDAN cohort**

| **Gene symbol** | ***P* value** | **Mean of Normal Tissue** | **Mean of CCA** | **Fold Change** |
| --- | --- | --- | --- | --- |
| HIST1H1E | 0.002655 | 1.882 | 2.969 | 1.577577046 |
| DCDC2 | 0.000968 | 6.964 | 9.181 | 1.318351522 |
| XRCC4 | 0.01019 | 6.201 | 7.094 | 1.144009031 |
| PRB1 | 0.342905 | 0.7609 | 0.8255 | 1.084899461 |
| APOBEC3C | 0.011859 | 8.372 | 9.008 | 1.075967511 |
| MAP2 | 0.167353 | 7.525 | 8.013 | 1.064850498 |
| H1F0 | 0.105669 | 11.18 | 11.56 | 1.033989267 |
| DNAJB5 | 0.534929 | 7.151 | 7.364 | 1.029786044 |
| NACA | 0.163824 | 12.71 | 12.91 | 1.015735641 |
| MSN | 0.573504 | 11.78 | 11.94 | 1.013582343 |
| TSR1 | 0.415091 | 8.958 | 9.073 | 1.012837687 |
| ZNF764 | 0.571364 | 7.236 | 7.315 | 1.010917634 |
| AARSD1 | 0.703465 | 6.522 | 6.592 | 1.010732904 |
| ZCRB1 | 0.531318 | 9.16 | 9.23 | 1.007641921 |
| SNX21 | 0.864024 | 7.906 | 7.936 | 1.003794586 |
| AKAP8L | 0.924662 | 9.433 | 9.45 | 1.001802184 |
| C1orf174 | 0.729918 | 8.156 | 8.113 | 0.994727808 |
| SRRT | 0.349752 | 10.74 | 10.61 | 0.987895717 |
| ARRB1 | 0.513619 | 8.683 | 8.544 | 0.983991708 |
| SRP19 | 0.178086 | 8.919 | 8.769 | 0.983181971 |
| NPPA | 0.731256 | 2.509 | 2.404 | 0.958150658 |
| YAF2 | 0.008296 | 8.438 | 8.082 | 0.957809908 |
| TMEM120A | 0.021986 | 10.08 | 9.608 | 0.953174603 |
| SFMBT2 | 0.147389 | 7.611 | 7.214 | 0.947838655 |
| CPNE8 | 0.017805 | 8.624 | 7.858 | 0.911178108 |
| NOSTRIN | 0.003089 | 8.554 | 7.383 | 0.86310498 |
| FAM13A | 0.00005 | 9.84 | 8.184 | 0.831707317 |
| KCTD14 | 0.017164 | 6.447 | 5.135 | 0.796494494 |
| CT45A3 | NA | NA | NA | NA |
| JHU04032 | NA | NA | NA | NA |
| Ncoa6(Mouse) | NA | NA | NA | NA |

**Supplementary Table 2**. TCGA cohort

| **Gene symbol** | ***P* value** | **Mean of Normal Tissue** | **Mean of CCA** | **Fold Change** |
| --- | --- | --- | --- | --- |
| DCDC2 | 8.331E-05 | 1.572 | 71.63 | 45.56615776 |
| HIST1H1E | 0.010287496 | 0.02744 | 1.032 | 37.60932945 |
| MAP2 | 0.005008166 | 0.364 | 4.665 | 12.81593407 |
| APOBEC3C | 6.1266E-05 | 1.965 | 19.83 | 10.09160305 |
| XRCC4 | 1.6781E-11 | 0.8349 | 4.312 | 5.164690382 |
| AARSD1 | 1.69244E-06 | 0.4536 | 2.053 | 4.526014109 |
| AKAP8L | 1.05181E-06 | 3.356 | 12.82 | 3.820023838 |
| TSR1 | 2.16922E-07 | 2.345 | 8.302 | 3.540298507 |
| MSN | 0.000792915 | 18.36 | 63.77 | 3.473311547 |
| SFMBT2 | 0.010892833 | 0.2269 | 0.7369 | 3.247686205 |
| NACA | 3.43273E-07 | 25.62 | 76.47 | 2.984777518 |
| SNX21 | 1.55446E-06 | 1.35 | 3.928 | 2.90962963 |
| SRRT | 5.39151E-09 | 9.267 | 26.04 | 2.809970864 |
| H1F0 | 0.00248832 | 64.49 | 173.7 | 2.693440844 |
| ZNF764 | 1.5562E-06 | 1.248 | 3.303 | 2.646634615 |
| ZCRB1 | 1.02958E-08 | 8.43 | 21.04 | 2.495848161 |
| DNAJB5 | 0.00537459 | 0.5571 | 1.327 | 2.381978101 |
| ARRB1 | 0.013332808 | 1.517 | 3.57 | 2.353328939 |
| NOSTRIN | 0.077989415 | 0.8615 | 1.913 | 2.22054556 |
| SRP19 | 5.70796E-07 | 2.839 | 6.048 | 2.13032758 |
| CPNE8 | 0.011279335 | 2.155 | 4.49 | 2.083526682 |
| KCTD14 | 0.247063208 | 0.7895 | 1.452 | 1.839138695 |
| NPPA | 0.476843346 | 0.07343 | 0.132 | 1.797630396 |
| YAF2 | 0.000141463 | 0.9647 | 1.6 | 1.658546698 |
| C1orf174 | 0.000850543 | 2.463 | 3.997 | 1.622817702 |
| TMEM120A | 0.584438276 | 30.45 | 33.2 | 1.090311987 |
| FAM13A | 1.04338E-10 | 4.029 | 0.8485 | 0.210598163 |
| PRB1 | 0.622633143 | 0 | 0.0005337 | NA |
| CT45A3 | NA | NA | NA | NA |
| JHU04032 | NA | NA | NA | NA |
| Ncoa6(Mouse) | NA | NA | NA | NA |
